# Supplementary material for: Strengthening health systems to improve the value of tuberculosis diagnostics in South Africa: A cost and cost-effectiveness analysis
Source: PLoS One. 2021 May 14;16(5):e0251547. doi: 10.1371/journal.pone.0251547 (PMC8121360; doi:10.1371/journal.pone.0251547)
Supplement: S1 Text — (DOCX) [file pone.0251547.s001.docx]

**S1 Text**

Supporting information for Foster et al. Strengthening health systems to improve the value of Tuberculosis diagnostics in high-burden settings: a cost and cost-effectiveness analysis.

Technical description of the model: a model of the relationship between healthcare worker behaviour and disease, explaining the transition of patients along the TB diagnostic and treatment pathway in South Africa.

# **Introduction**

Tuberculosis (TB) is a leading cause of mortality in South Africa, and is associated with poor socio-economic status (1), nutritional deficiencies (2,3), in additional to co-morbidities such as HIV and Diabetes (4,5).

People living with HIV (PLHIV) have both a higher probability of developing active TB, and once ill with tuberculosis will have a higher rate of mortality. Diagnosing tuberculosis in PLHIV is complicated by the disease dynamics. Patients who are HIV positive, are more likely to develop extra-pulmonary TB and in those with pulmonary TB, there is less cavitation in the lungs with pauci-bacillary sputum samples making it harder to diagnose the disease when using sputum-based modalities such as sputum smear microscopy and Xpert MTB/RIF. More recently, urine-based TB diagnostic tests have been shown to reduce mortality in hospitalised, high-risk PLHIV, with low CD4 counts (6). In contrast, while PLHIV have a higher TB mortality rate, those who are HIV negative are currently thought to be more likely to contribute to TB transmission in the community (7), due to cavitation in the lungs with bacilli expelled when breathing or coughing.

In 2012, South Africa adopted the Xpert test as a replacement test for sputum smear microscopy. Initial mathematical models predicted that the improved sensitivity of Xpert compared to smear microscopy would lead to a reduction in TB-associated mortality at a population level. However, empirical studies conducted during the roll-out of Xpert showed that the implementation of the new diagnostic guidelines did not lead to significant reduction in mortality (8–10). The range of available TB diagnostics therefore remain inadequate to effectively identify TB and reduce mortality.

Mathematical models have been extensively used to estimate the prevalence of TB in a population; used to simulate interactions between disease processes and biomedical interventions; to better understand interactions between people facilitating transmission or between populations and biomedical interventions. More recently, mathematical models have been used to simulate patients’ interactions within a health system for the estimation of resource needs at the population level (11–14). However, most of these studies used observational data in the form of routinely collected data or secondary data from a variety of discrete studies, studying different populations within their context. There was therefore a need to understand, given a specified cohort, and with evidence on the pragmatic empirical data, how patients move through the patient care pathway and how this relates to their health outcomes.

# **Mathematical models of TB diagnostic processes**

The majority of the model-based cost-effectiveness analyses of diagnostics were conducted from the provider perspective (12,13,15–19), with the exception of a study conducted in Tanzania (12), as well as the within-trial cost-effectiveness analysis as part of this XTEND trial (20) where a patient perspective was also considered. None of the model-based analysis, however explicitly included the limitations of sputum-based diagnostic modalities in their analyses. The additional value of this model-based analysis however rests on extensive primary cohort data collected, the rigour of the evaluation of the trial cohort, as well as the range of perspectives, both provider and patient included in this analysis.

# **Model structure**

Mathematical models that have been developed to investigate the value of TB diagnostics, can be classified as either disease (transmission) models that represents the macro-level interactions associated with how people in populations move through health states associated with the disease or economic models. A challenge with disease models is that it is often difficult to represent the smaller time step processes associated with diagnostics at the level of detail required for an economic evaluation, though some have proposed possible approaches for collapsing these states and representing this parsimoniously (21). In contrast, economic models focus on the interaction between resource use and patient outcomes, either at a patient-level (individual-level) or at a cohort level. Cohort-level economic models estimates the expected costs and outcomes across a specified population.

The model structure was defined by a set of mutually exclusive HIV disease states and subdivided to account for 1) TB/HIV co-infection and progression of TB disease, 2) diagnostic and treatment algorithms, 3) drug resistant TB strains. The model structure is shown in Figure S**3** and Figure S**4**. To accurately present the complexity of TB diagnostic guidelines, transition sub-trees were added to the symptomatic Markov nodes (22). The sub-trees also allow for patient’s prior history to be considered, mediating the ‘Markovian assumption’ or the well-known tendency of Markov models to be “memoryless” (23). The model simulates individuals’ movements between health states deterministically in defined (monthly) cycles, following a cohort from the start of TB symptoms to death or resolution of symptoms. It is possible for an individual to remain in each state in successive time periods, or to move between health states (and disease characteristics) monthly, dictated by state transition probabilities, and in the initial states, half cycle corrections were used to adjust for timing in the model. Absorbing states have no transition subtree or affiliated jump state and therefore the probability of exiting the state is zero. The model was developed and implemented using TreeAge Pro software. The model follows on earlier TB economic models but with a focus on explicitly modelling the interactions between patient pathways and the patient-important outcomes of the challenges experienced in health systems (11,12,17,24–26).

HIV/TB co-infection alters the rate of progression of TB disease but also dictates the sensitivity of the diagnostic tests and, due to low bacillary load, reduces the ability to diagnose TB from a sputum sample. Three subcategories were created to account for the differences in disease progression, utilisation, diagnostic - and treatment algorithms of those, HIV negative, HIV positive not on ART, and HIV positive on ART. Patients in the model are therefore at any time categorized by the combination of their TB and HIV (on or off ART) status. Each of the five core health states, namely TB symptoms, out of care, Drug Sensitive (DS) treatment, Retreatment, and Drug Resistant (DR) treatment are replicated for each of six possible HIV and TB categories namely HIV negative no TB, HIV negative with TB, HIV positive no TB, HIV positive with TB, HIV positive on ART no TB, and HIV positive on ART with TB. These states are then expanded into 81 unique health states in the model. Patients’ transition between model states and subdivisions based on transition probabilities. Where transitions over time did not occur at a constant rate (time-varying), tunnels linking to parameter tables as well as cost curves were used to represent the change over time in the model. It is possible, within the model, for an individual to transition from being HIV positive to HIV positive on ART, however, within the model, we do not include HIV transmission or diagnosis in the model as a simplifying assumption.

The model structure is shown in Figures 1 and 2. All individuals enter the model as symptomatic; receive an initial diagnostic test, followed by additional tests and/ or treatment. If the individual does not start treatment within the first month of a diagnostic test result, they will move to the ‘out of care’ box in the model and return to treatment at a rate estimated from the empirical data.

Each health state is associated with an estimated resource use, costs and a disability disutility based on the group’s TB and HIV disease profile. The model is used to estimate the mortality, average cost and Disability Adjusted Life Years (DALYs) averted from time in health states to firstly, the end of a single TB episode and secondly, to end of life. TB and HIV- associated mortality was sourced from secondary published sources in South Africa and calibrated to the ‘within trial’ observed mortality rate. Disability-weights were derived from the global burden of disease study. All future costs and benefits included in the study, were discounted at 3%, and varied between 0 and 10% in the sensitivity analysis.

Figure 1. Outline of the Markov cycles of the model structure


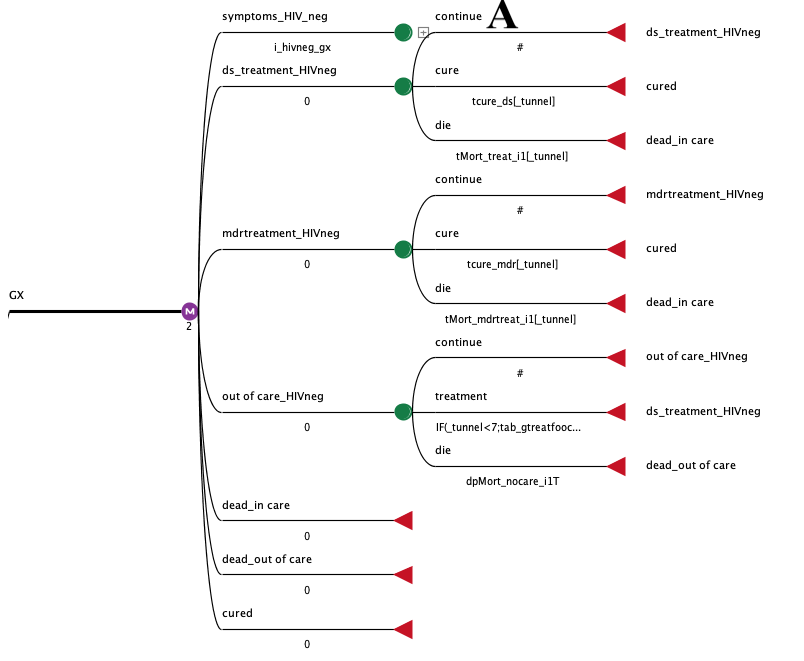


In this figure, the same structure shown for HIV negative patients without TB, is repeated for patient groups who are (2) HIV negative no TB; (3) HIV positive with TB; (4) HIV positive no TB; (5) HIV positive on ART no TB; (6) HIV positive no TB. Where nTB refers to no TB; rr treatment refers to Rifampicin resistance, leading to multi-drug resistant TB (MDR-TB) treatment; and ds treatment refers to drug-sensitive treatment. GX refers to Xpert MTB/RIF.

Figure 2. Decision tree structure, representing the diagnostic processes of the model.


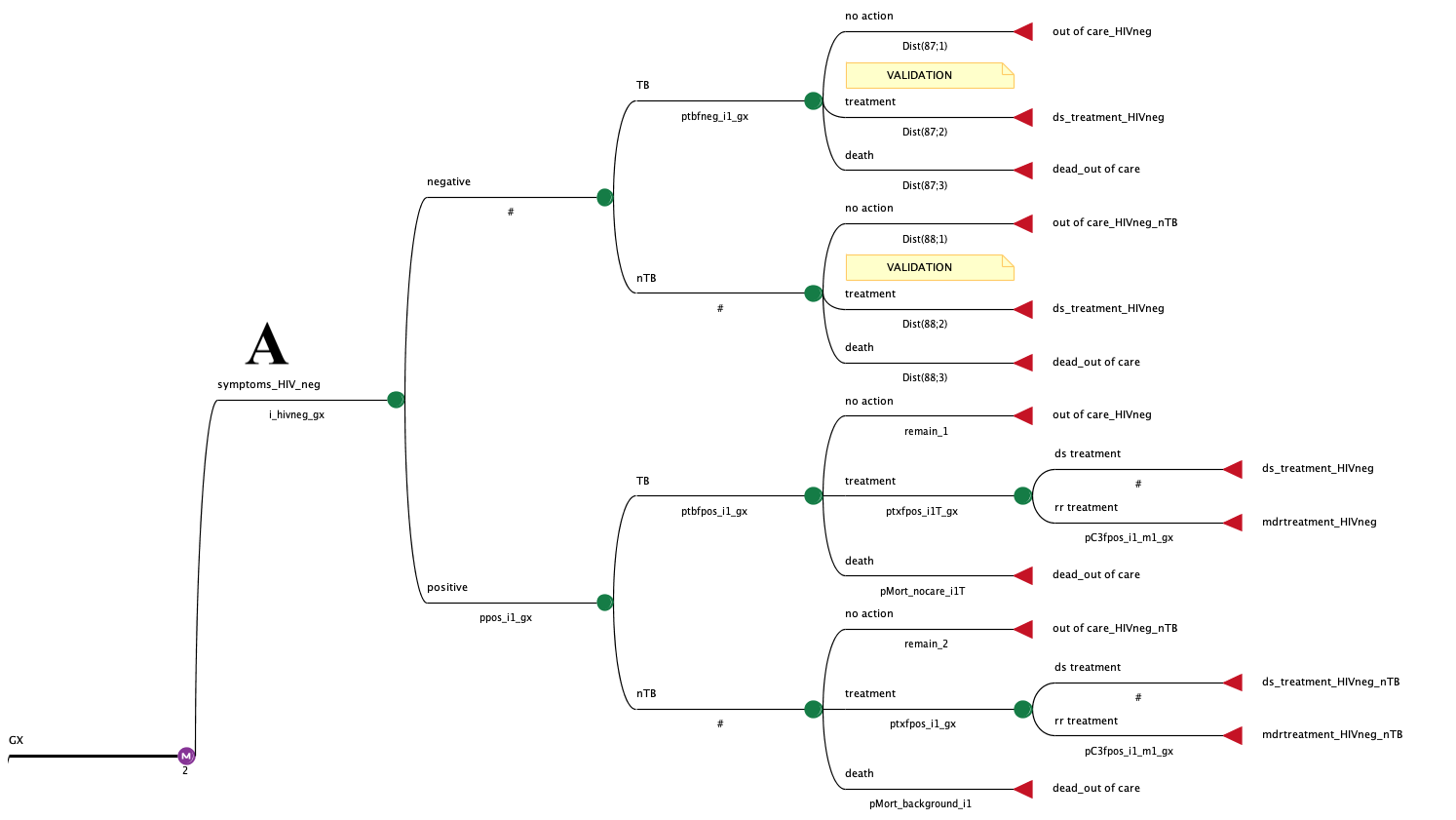


Where nTB refers to no Tuberculosis; rr treatment refers to treatment for Rifampicin resistance leading to MDR-TB treatment; and ds treatment refers to drug-sensitive treatment. The variables below the line of the tree structure denotes the name of the parameter driving movement through the model. The structure shows the part of the model where patients enter the model on the left, move through the health and process states and the red triangle at the end of the branch represents a movement to the next Markov cycle and state. The states transitioned to are shown in Figure 1*.*

Outcomes from treatment include treatment failure, cure and death. Cure is assumed to occur at six months on treatment if no treatment failure and still on drug-sensitive treatment; nine months on retreatment and 24 months on multi-drug resistant treatment. A proportion of drug resistant TB patients who are not cured, continue treatment until death.

# **The XTEND trial and population modelled**

XTEND was a pragmatic cluster randomised control trial, embedded in phase three of the national roll-out of Xpert in South Africa. Clusters were defined as a laboratory with two affiliated healthcare clinics. Twenty laboratories were selected from four provinces, and randomised to Xpert (immediate implementation) or microscopy (deferred Xpert implementation) study groups (8). Patients were eligible if older than 18, not on TB treatment, resident in the area for the next eight months, and if clinic staff requested a sputum specimen to investigate for possible TB. Patient management was conducted by clinic staff in line with routine practice. Participants were enrolled into the study and interviewed again six months later to determine whether and when TB treatment or anti-retroviral therapy (ART) was started. Deaths were recorded through reports from clinic staff, participant-nominated contacts, and by accessing the Department of Home Affairs vital statistics database.

The empirical data used in this study to parameterise the population being modelled were collected as part of the XTEND study. The provinces represent very rural settings such as those in the Eastern Cape and highly urban settings such as found in Gauteng.

The epidemiological burden and health system indicators relating to TB and HIV care in South Africa are summarised, by province, in Table 1. The provinces from which the XTEND study was sampled are highlighted in the table.

Table 1. Programmatic, epidemiological and health service indicators at the time of the XTEND study (2012 – 2014), by province in South Africa


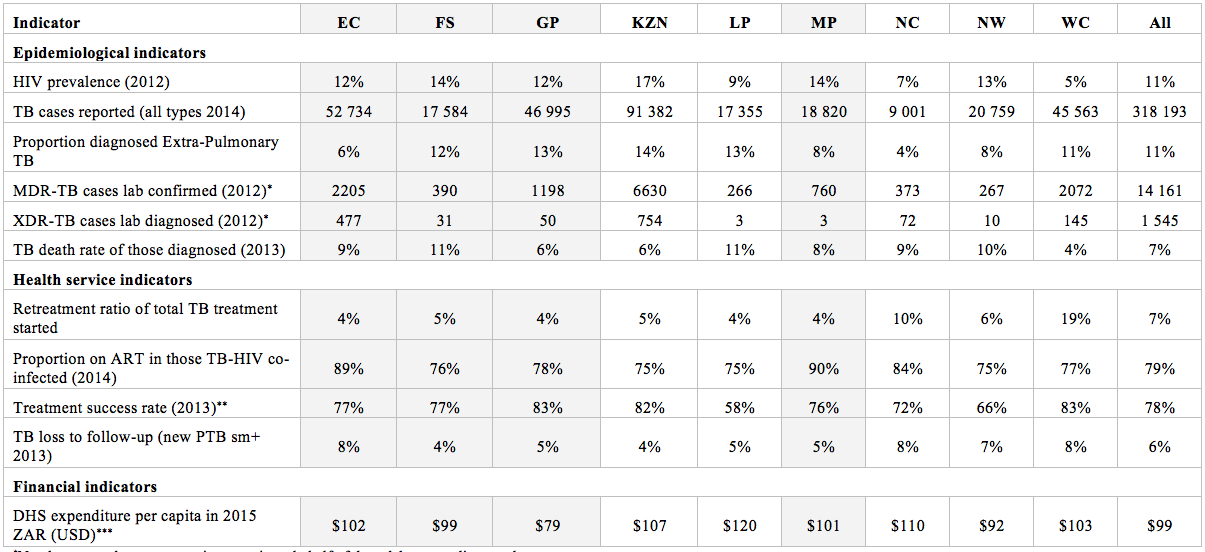


^*^Numbers started on treatment is approximately half of those laboratory diagnosed.

^**^Treatment success rated defined as the ‘the proportion of all types of TB patients that were either cured or completed a full course of treatment.

^***^Average 2015 ZAR/USD exchange rate (1 USD = ZAR15,19), from <http://www.oanda.com/currency/historical-rates/>.

DHS: district health system; EC: Eastern Cape; FS: Free State; GP: Gauteng; KZN: KwaZulu Natal; LP: Limpopo; MP: Mpumalanga; NC: Northern Cape; NW: North West; WC: Western Cape. (Source: 2014/2015 Health Systems Trust District health barometer: 228; STATS SA publication P0302, P03022015: 7; HSRC 2012 SA national HIV prevalence, incidence and behaviour survey).

The models of TB service varied between the facilities sampled in the XTEND trial, some of the main differences are detailed in the supplementary appendix of Foster et al. (27). The annual clinic headcount ranged between 14 887 at one rural facility in a sparsely populated area in the Eastern Cape, and 120 589 at a busy clinic on the outskirts of the capital. TB treatment was offered as daily facility-based DOTS at some facilities, two-weekly collection at the facility in others, while other facilities used a largely community-based DOTS system. At a facility sampled in a rural agrarian community, patients living and working on farms were given a monthly supply of treatment.

Table 2. Summary service statistics related to the model of TB service in the facilities sampled in the XTEND study.

|  | **Intensive phase visits in new treatment, median (range)** | **Intensive phase visits in retreatment, median (range)** | **Continuation phase visits in new treatment, median (range)** | **Continuation phase visits in retreatment, median (range)** |
| --- | --- | --- | --- | --- |
| Eastern Cape |  |  |  |  |
| facility 1 | 4 (2; 5) | 18 (4; 28) | 4 (2; 4) | 4 (4; 4) |
| facility 2 | 2 (2; 3) | N/A | N/A | N/A |
| facility 3 | 0.5 (0.5; 1) | N/A | 2 (2; 3) | N/A |
| Free State |  |  |  |  |
| facility 4 | 4 (4; 24) | N/A | 1 (1; 1) | N/A |
| facility 5 | 1.5 (1.5; 2) | N/A | 2 (2; 2) | N/A |
| facility 6 | 20 (2; 20) | N/A | 0.5 (0; 4) | N/A |
| Gauteng |  |  |  |  |
| facility 7 | 17 (4; 23) | 21 (16; 27) | 4 (4; 17) | 13 (4; 22) |
| facility 8 | 1 (1; 1) | N/A | 1 (1; 1) | N/A |
| Mpumalanga |  |  |  |  |
| facility 9 | 1 (1; 1) | 0.5 (0.5; 0.5) | 2 (1; 2) | N/A |
| facility 10 | 1 (1; 1.25) | 2 (2; 2) | 2 (2, 2.5) | 2 (2; 2) |

In the Table, data collected from clinic managers between July 2011 and June 2012 are summarised.

# **Patient pathways through care**

Clinical guidelines are used as a set of recommended rules for practice in clinical settings. These rules of practice are typically part of an evidence synthesis process to determine the most effective or preferred manner of identifying and treating patients with specified conditions.

## **5.1 Diagnostic algorithms**

The South African TB diagnostic and treatment guidelines implemented at the time of the XTEND study are described below. At the time of the study, TB screening was primarily passive in that it depended on patients with symptoms suggestive of TB presenting to the health facility seeking care, however there was a move towards approaches to more intensified TB case finding. In the interim, however, the South Africa TB programme has extensively scaled up TB case finding in health facilities.

- TB screening: primarily passive and depends on self-presentation of persons with TB symptoms to health care facility. Following the TB investment case, there has been a shift in these recommendations towards more active TB case finding.
- Following screening, the TB diagnostic algorithm at the time of the study is presented in Figure 1 and Figure 2.
- With regards to TB treatment, following the introduction of Xpert, the extended treatment regimen used in patients who had a prior episode of TB (retreatment) was replaced by the standard regimen for drug sensitive TB treatment if no evidence of Rifampicin resistance, irrespective of previous TB episodes.
- The standard (drug-sensitive TB) treatment regimen is RHZE daily for 2 months (intensive phase); followed by RH daily for 4 months (continuation phase). In the case of extra-pulmonary TB, the continuation phase is prolonged to 7 months. It is also recommended that patients are started on Vitamin B6 (25 mg daily).
- If drug resistant, INH mono-resistant TB is diagnosed - RHZA for 6 to 9 months; any Rif resistant TB - MDR-TB regimen for 18 to 24 months.
- All patients diagnosed with Xpert, will also need to have a baseline smear and be monitored using smear microscopy. If smear negative or EPTB, will be monitored clinically. A smear microscopy is done a week before the end of the intensive phase of treatment. If the smear microscopy is positive conduct a LPA (or culture and DST) to check drug sensitivity. Repeat one week before the end of the continuation phase. If positive, patient is identified with treatment failure. Re-check patient’s drug susceptibility.
- Anti-retroviral therapy: ART initiation if TB diagnosed before starting ART, if a patient has a CD4 count < 50 start ART straight away. If the CD4 count > 50 start ART before end of intensive phase. If a patient develops TB while on ART, continue treatment but adjust the ART dosages to minimise drug-drug interactions.
- Directly Observed Therapy (DOTS), the recommendation in the NTCP adult TB guidelines is that patients’ treatment monitoring should be adapted in a way such as would most suit the patient.

While the summary, in Figure 1 and Figure 2 presents the official guidelines, these guidelines were not uniformly implemented across all nine provinces in South Africa. For example in the Western Cape instead of a single sample, two samples were taken consecutively at the health facility and sent to the laboratory (28). This allowed for an additional sample to be available in the case the first sample was not viable (low bacillary load or saliva as opposed to sputum) or if there is the need to culture a sample to check for drug resistance. A single sample is not reusable after an Xpert test has been performed, given that the bacteria is destroyed in the sample as part of performing the test. Routinely taking two sputum samples, is therefore thought to reduce the need to request another sample from the patient, which would greatly delay diagnosis.

Figure 3. Xpert-based TB diagnostic algorithm.


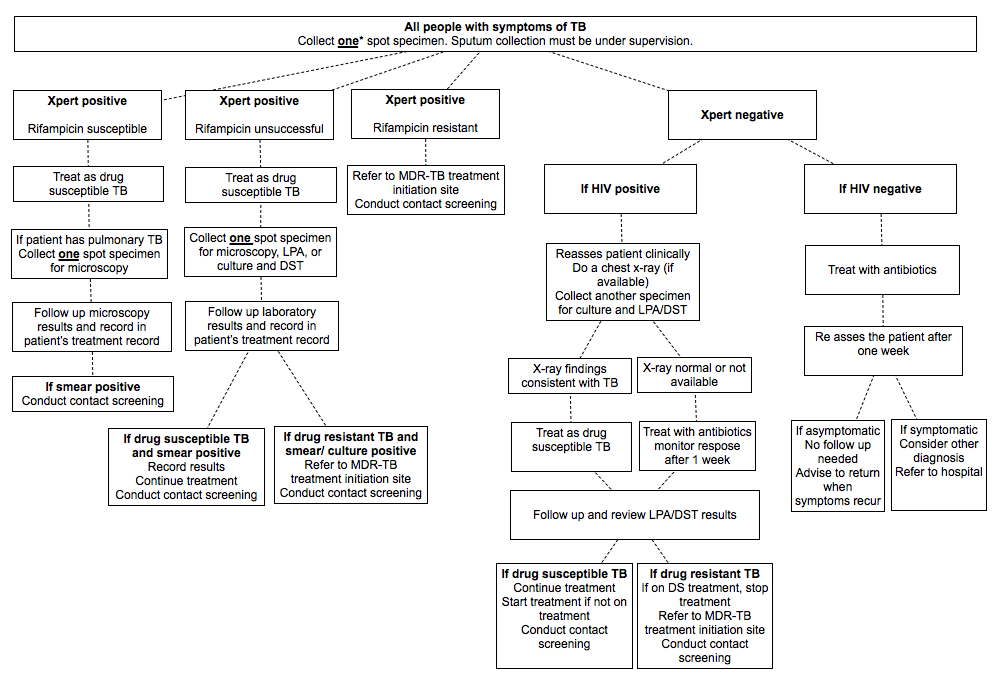


In the figure, DST refers to drug-sensitive treatment; MDR refers to multi-drug resistant treatment; X-ray refers to a chest x-ray.

Figure 4. Microscopy-based TB diagnostic algorithm.


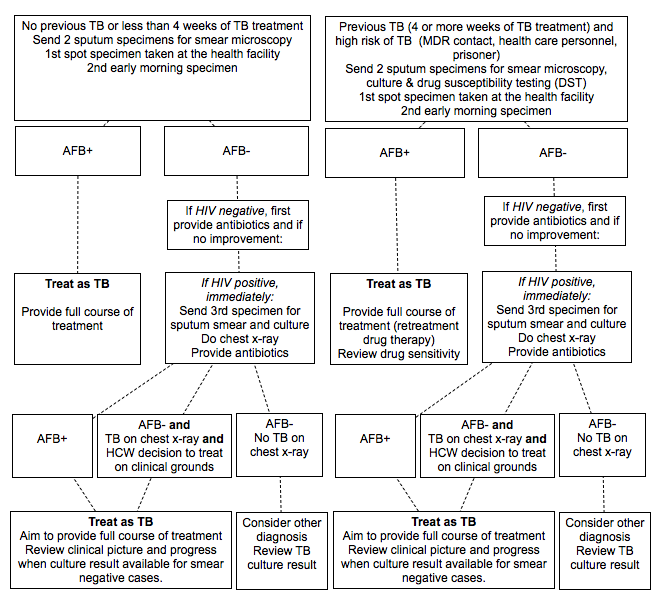


In the figure, AFB refers to Acid-Fast Bacilli; and HCW speaks to healthcare worker.

Cumulative distribution functions, representing how patients in the cohort moves from out of care to treatment, were estimated for each individual type.

In the smear arm of the study, the probability of starting treatment over time for those HIV negative was [0.0; 0.0; 0.0; 0.0; 0.0;0.0; 0.0]; HIV negative with TB [0.0; 0.282; 0.205; 0.083; 0.085; 0.0; 0.0]; HIV positive [0.0; 0.0; 0.0; 0.0; 0.0; 0.0; 0.0]; HIV positive with TB [0.0; 0.150; 0.060; 0.050; 0.030; 0.0; 0.0]; HIV positive on ART [0.0; 0.0; 0.0; 0.0; 0.0; 0.0; 0.0]; HIV positive on ART with TB [0.0; 0.21; 0.09; 0.09; 0.05; 0.0; 0.0].

## **5.2 Empirical estimation of pathways through care**

Model parameters were estimated by calculating conditional probabilities from patient-level cohort data collected as part of the XTEND study. Probabilities were estimated for the specified patient types, 1) HIV positive, 2) HIV positive on ART and 3) HIV negative., for each health state by patient type and time step. For each transition probability, the mean and standard deviation was estimated. Distributions for binomial events such as transition probabilities and disability weights were implemented in the model as Beta distributions. Where there are multiple probabilities around a decision node, Dirichlet distributions were used so as not to exceed one during sampling (29). Given the skewed nature of cost data and utilisation, with a wide variation in values between provinces, Gamma distributions were used.

# **Validation and calibration of the model**

Structural uncertainty in the model was minimised by validating the outcomes of the mathematical model to the statistical analysis as part of an iterative process that led to a refinement of the model architecture. This process allowed for the identification of possible model programming errors, as well as to identify differences in assumptions between the two analyses.

Adjusting for baseline differences between the study arms

The model was adjusted for the imbalance in patient population between the two arms of the trial, by making the following changes:

- remove calibration modification (50% mortality reduction for those HIV positive) for healthier patients in the Xpert arm
- same proportion of HIV and ART types of individuals through the model
- recalculate (using the 2x2 tables) the probabilities of having a positive test result and starting treatment based on an equivalent bacteriologically confirmed TB prevalence between the two arms

Calibration approach

The possible parameter combinations and values to identify the best possible fit of the time to death and time to treatment curves; and relevant statistics are presented in Table 6.2.1. The difference between the curve and the trend line was determined by plotting the empirical data. The fit of the curve was expressed using three metrics: the maximum error (ME), the square root of the error (SqE) and the number of points

While model validation is the process of comparing the outcomes of the model to observations, calibration refers to the iterative process of varying the values of unobserved variables until the outcomes of a model fits the outcomes being fitted to (30). A strength of this study is the use of detailed empirical data, which include transitions through care pathways and resource use, from a single cohort.

The challenge with calibrating decision analysis models relates to the fact that these models typically model micro-processes, where there is a need for greater precision when estimating the resource utilisation and resulting costs of an intervention (31,32). The trade-offs during calibration and validation is that if the values are fitted to many narrowly specified calibration ranges, the model may be overfitted and not be as representative of the system it is trying to emulate when extrapolating from the available data (33). Using measures to consider the overall fit of the curve as opposed to a fitting to a single point, mediates this challenge to some extent as places the focus on patterns of behaviour as opposed to a single statistic.

We calibrated the original model structure to both arms (smear microscopy as well as Xpert) of the trial, in order to refine the structure and explore covariates that drive the differences between the study arms in more detail. During the analysis, the model is then restricted to the Xpert arm only, with supportive investments.

Given the pragmatic nature of the trial whereby there was no interference with public facilities’ current practice, the gold standard for TB diagnosis, culture was not artificially requested for each patient (culture for all was not part of the South African TB diagnostic guidelines at the time of the study). Therefore, given that neither smear microscopy nor Xpert is likely to identify every patient with TB, we were unable to empirically estimate what proportion of those with a negative diagnostic test result were correctly started on treatment i.e. the sensitivity and specificity of healthcare workers’ intuitive judgement to start people on treatment without a definitive TB test result. The calibration was used to estimate the best combination of values to allow for a good fit of the model to the empirical data. The unobservable parameters in the model included 1) the effectiveness of clinical decision-making to correctly start those with TB on treatment from a negative test result and 2) the effectiveness of clinical decision making in the diagnostic algorithm in starting those with TB correctly on treatment. We therefore calibrated, through a sequential, iterative process, the mortality curve generated from the model output, to the Kaplan-Meier mortality curve from the trial. In a similar process, we calibrated the time-to-treatment curve from the model output to the Kaplan-Meier time-to-treatment curves from the trial. The best fit was deemed the configuration of behaviour that provided the best fit of both the mortality and treatment curves simultaneously.

To derive this from trial observed mortality (up to six months after a diagnostic test) and measured health care worker behaviour and outcomes, we populated our model with mortality rates from secondary data, shown in Table 11. Figure 13 shows an example of how the 2x2 table was used to estimate the Positive – (PPV) and Negative Predictive Values (NPV) of the diagnostic processes.

The probability of bacteriologically confirmed TB from a positive or negative TB test result (the positive – or negative predictive value of the test) was estimated based on the reported sensitivity and specificity of the test, and the number of positive test samples in the trial (31,32). The approach is demonstrated below, using the example of Xpert arm of the study. The same approach is then repeated to estimate the probability of TB if test positive p(TB|test positive) and the probability of TB if test negative p(TB|test negative).

For the *Xpert arm of the model*, we used the pooled sensitivity 0,860 in those HIV negative and 0,790 in those HIV positive and those HIV positive on ART as estimated in the meta-analysis (32). A specificity of 0,99 was used.


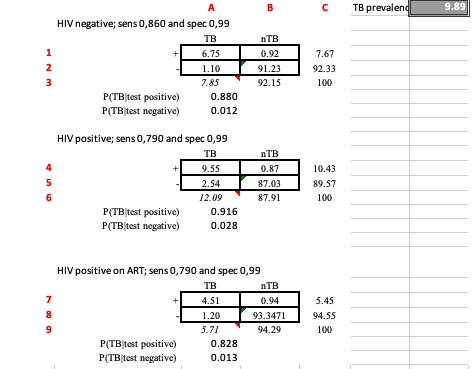


Figure 5. Xpert unadjusted 2x2 table.

A3 in Figure 13, TB prevalence, was estimated using the sensitivity and specificity of the test, solving for x in the following equation:

0.86x + 0.01(100-x) = 7.67

0.86x + 1 – 0.01x = 7.67

0.86x – 0.01x = 6.67

0.85x = 6.67

x = 7.85 which relates to cell A3

A1 was then estimated by multiplying the sensitivity of the test by A3.

C1 was estimated by dividing the number of positive over the total sample (from the dataset).

The P(TB|test positive) = TB+/ Allpos

The P(TB|test negative) = TB-/Allneg

For the *sputum smear microscopy* arm of the model, we used the pooled sensitivity of fluorescent microscopy calculated in a meta-analysis; 0,723 in those HIV negative and 0,446 in those HIV positive (34). In those HIV positive on ART, we used the same sensitivity as the general HIV positive value provided. A specificity of 0,999 was used.

The TB prevalence in each of the samples, was unknown. TB prevalence was therefore estimated using the same approach as described above and estimated a prevalence of 13.19%.

Given that the population modelled was the microscopy arm of the trial, we re-estimated the probabilities of TB from test positive and test negative for a population with a TB prevalence of 13.19%. The revised probabilities were:

**HIV negative**

P(TB|test positive) = 0.877

P(TB|test negative) = 0.012

**HIV positive**

P(TB|test positive) = 0.936

P(TB|test negative) = 0.038

**HIV positive on ART**

P(TB|test positive) = 0.938

P(TB|test negative) = 0.039

From the probability of TB after the test result, we needed to estimate the probability that health care worker’s decision-making after a negative test result was either correctly identifying TB or not. In other words, whether health care workers were correctly starting patients with TB on treatment after a negative test result, averting associated mortality and whether their decision to provide additional testing after a negative test result was correctly identifying TB or not. Starting TB treatment earlier, would reduce TB associated mortality if correctly started on treatment.

The initial distribution of the probability to start TB treatment after an initial negative test result was estimated using the following equation:

|  | N*_i,t_* = A*_i,t_*R + B*_i,t_*W | (2) |
| --- | --- | --- |

Where: N is the probability of a health care worker acting as if the patient has TB (either by ordering further diagnostic tests or by starting treatment based on clinical suspicion), after a negative test result, where further diagnostics are not ordered at time *t* and for individual type *I*; A is the probability of true TB at time *t* and for individual type *i* with this decision being the correct decision i.e. true TB represented by the letter R; B is the probability that the patient does not have TB at time *t* and for individual type *i* with this diagnostic decision therefore being incorrect presented by the letter W.

We started the calibration, initially assuming that health care workers had an equal chance of correctly and incorrectly identifying someone as having TB, no difference between the decisions to start people on treatment from a negative test result and doing additional tests for the diagnostic negative pathway, the impact of the change in probability on the predicted mortality in the model was sequentially calculated and matched against the Kaplan-Meier mortality curve from the trial, see Figure 14 and Figure 15.


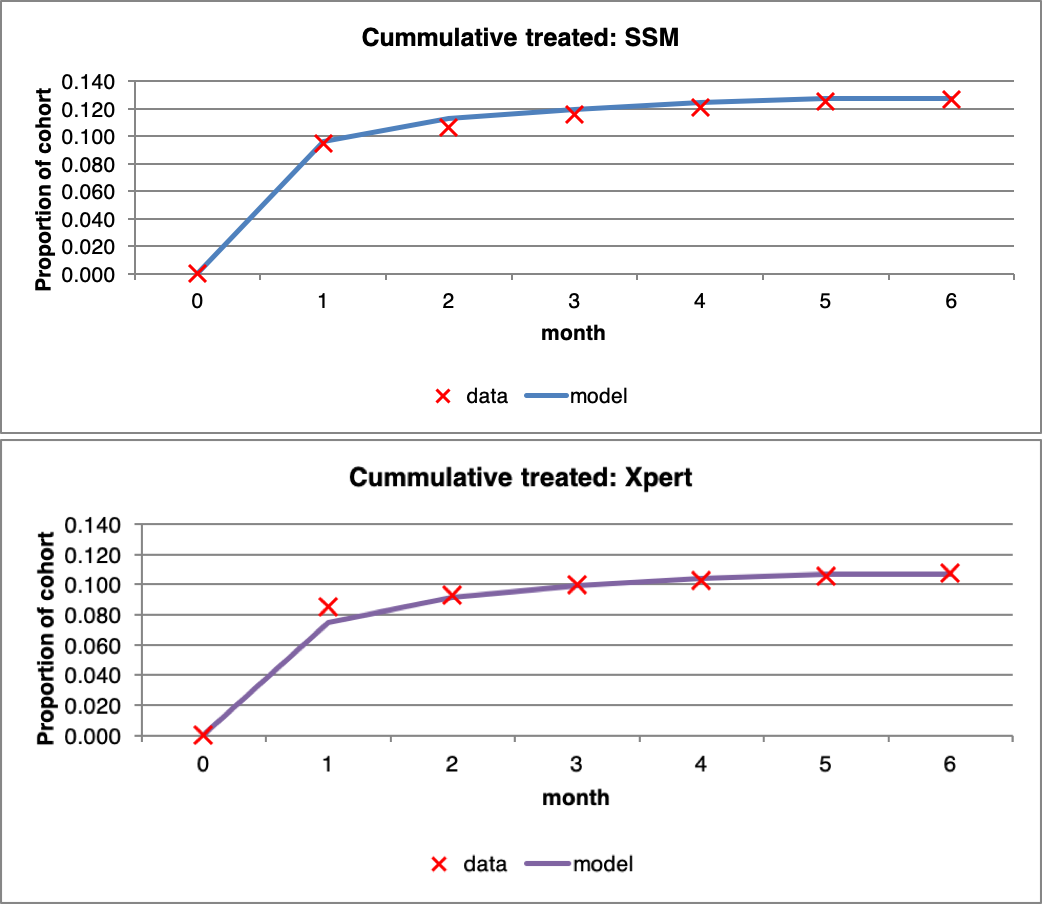


Figure 14. Comparing the fit of the time-to-treatment curves from the empirical data to the modelled estimates.

The graph shows the cumulative proportion of the modelled cohort at monthly intervals. The model estimates of the time to starting treatment is represented by the solid line, with data points from the cohort study is presented as red crosses at each of the monthly time points. Two graphs were generated, one representing the sputum smear microscopy arm of the cohort; and the other the arm of the study that were randomised to receive a Xpert test.


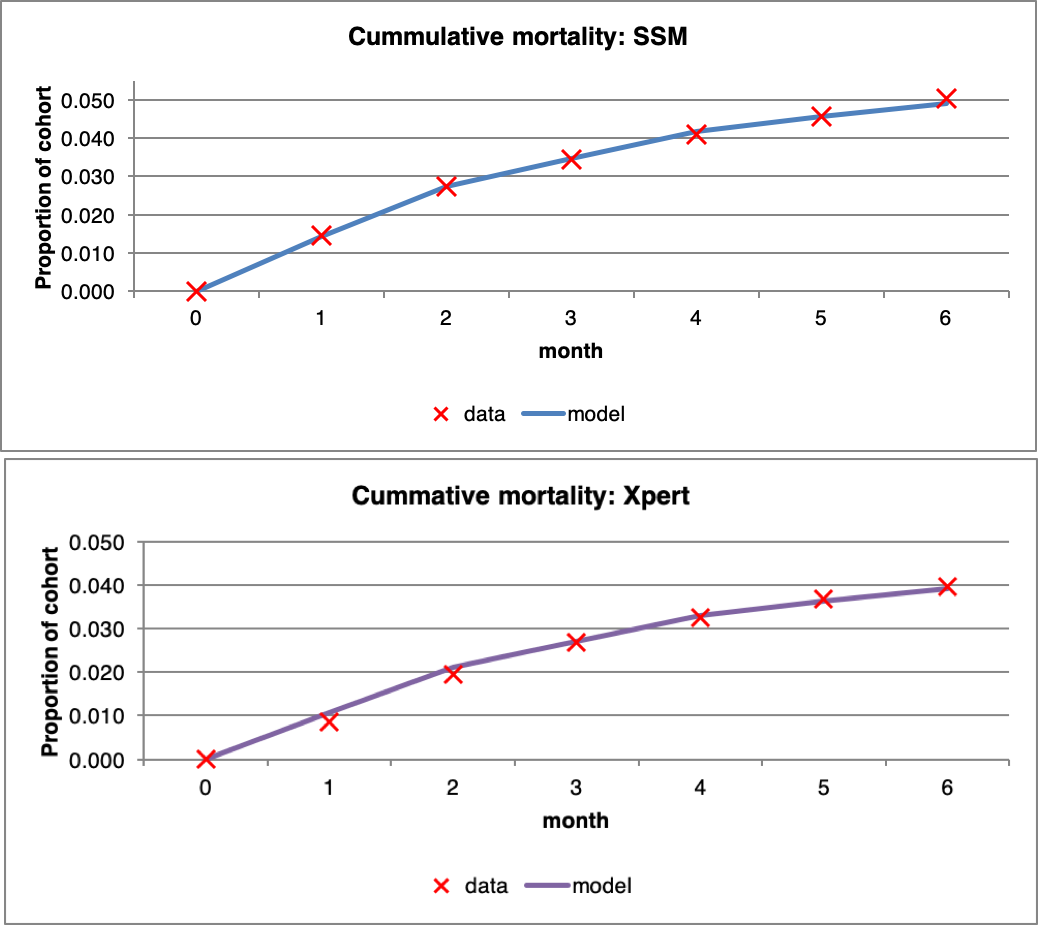


Figure 15. Comparing the fit of the time-to-death curves, model outputs to the data.

The graph shows the cumulative proportion of the modelled cohort at monthly intervals. The model estimates of the time to death is represented by the solid line, with data points from the cohort study is presented as red crosses at each of the monthly time points. Two graphs were generated, one representing the sputum smear microscopy arm of the cohort; and the other the arm of the study that were randomised to receive a Xpert test.

The probability of starting treatment from a negative test result or from the negative pathway was estimated from the empirical data. Given the use of conditional probabilities to drive movement through the model, the calibration equations resulted in adjustments needed in other parts of the model, such as the denominator for treatment from out of care. These were calculated in a populated Excel spreadsheet. For each of the three uncertain parameters, we sequentially calibrated the probability to fit the mortality curve against the trial data observed in the trial, both in size and shape of the curve over time. With each subsequent calibration, the effect on related variables was re-estimated and repopulated in the appropriate model parameters.

Table 4. Measures of model fit.

|  | **Smear microscopy** | | | | | | **Xpert MTB/RIF** | | | | | |
| --- | --- | --- | --- | --- | --- | --- | --- | --- | --- | --- | --- | --- |
|  | **ME_mort** | **ME_treat** | **SqE_mort** | **SqE_treat** | **points_mort (<5)** | **points_treat (<20)** | **ME_mort** | **ME_treat** | **SqE_mort** | **SqE_treat** | **points_mort (<5)** | **points_treat (<20)** |
| RW1_EPTB0% | -42 | -200 | **55** | **1143** | 6 | 6 | 8 | 158 | **5** | **694** | 6 | 0 |
| RW1_EPTB2% | -5 | -183 | **5** | **953** | 6 | 6 | 28 | 167 | **26** | **780** | 3 | 0 |
| RW1_EPTB4% | 16 | -166 | **18** | **780** | 4 | 6 | 48 | 177 | **73** | **870** | 1 | 0 |
| RW1_EPTB6% | 37 | -148 | **58** | **625** | 3 | 6 | 68 | 186 | **147** | **964** | 0 | 0 |
| RW1_EPTB8% | 58 | -131 | **127** | **489** | 2 | 6 | 88 | 196 | **247** | **1064** | 0 | 0 |
| RW0.5_EPTB0% | -23 | -154 | **17** | **690** | 6 | 6 | 10 | 175 | **6** | **854** | 6 | 0 |
| RW0.5_EPTB2% | -2 | -140 | **5** | **569** | 6 | 6 | 31 | 179 | **31** | **893** | 3 | 0 |
| RW0.5_EPTB4% | 19 | -127 | **23** | **461** | 4 | 6 | 52 | 183 | **84** | **933** | 0 | 0 |
| RW0.5_EPTB6% | 40 | -113 | **69** | **365** | 2 | 6 | 73 | 187 | **167** | **973** | 0 | 0 |
| RW0.5_EPTB8% | 62 | -99 | **146** | **281** | 2 | 6 | 93 | 191 | **277** | **1015** | 0 | 0 |
| RW0.25_EPTB0% | -22 | -131 | **16** | **506** | 6 | 6 | 11 | 184 | **7** | **941** | 6 | 0 |
| RW0.25_EPTB2% | -1 | -119 | **6** | **415** | 6 | 6 | 32 | 185 | **34** | **953** | 3 | 0 |
| RW0.25_EPTB4% | 21 | -107 | **25** | **333** | 4 | 6 | 53 | 186 | **90** | **965** | 0 | 0 |
| RW0.25_EPTB6% | 42 | -95 | **75** | **261** | 2 | 6 | 75 | 187 | **177** | **977** | 0 | 0 |
| RW0.25_EPTB8% | 64 | -83 | **155** | **199** | 2 | 6 | 96 | 189 | **294** | **990** | 0 | 0 |

In the Table, each of the values represents the arithmetic mean drawn across 1 000 model samples. ME refers to the marginal error and SqE represents the square root of the marginal error. The columns labelled points represents the number of points in the time-series where the model results are acceptably close to observations (within 5 or 20 units). Mort signifies that mortality is the outcome, while treat represents treatment as the outcome compared.

The difference between the curve and the trend line was determined by plotting the empirical data. The fit of the curve was expressed using three metrics; the maximum error (ME), the square root of the error (SqE) and the number of points in the time-series where the model results are acceptably close to observations (within 5 or 20 units).

ME_mort represents the maximum error which is the sum of the differences between model estimate of mortality and the value observed in the trial and is to be minimised; ME_treat is a similar statistic but refers to the proportion of those in the cohort who starts treatment; SqE_mort refers to the mean square root of the error or the difference between the observed and modelled estimates of mortality and treatment started (SqE_treat); points_mort and points_treat is a measure of the number of points modelled that are within in the first instance five units, followed by 20 units from the point observed from the trial. This measure therefore speaks to the fit of the trend. To find the best fit for the combined curves representing the relationships between treatment started and mortality reduction, the squared error of mortality was plotted against the squared error of treatment, summarised in Table 3, and shown graphically in Figure 16. The plot closest to the left and lowest on the *y* axis corresponds to the plot that represents the best fit of the interaction between mortality and TB treatment.


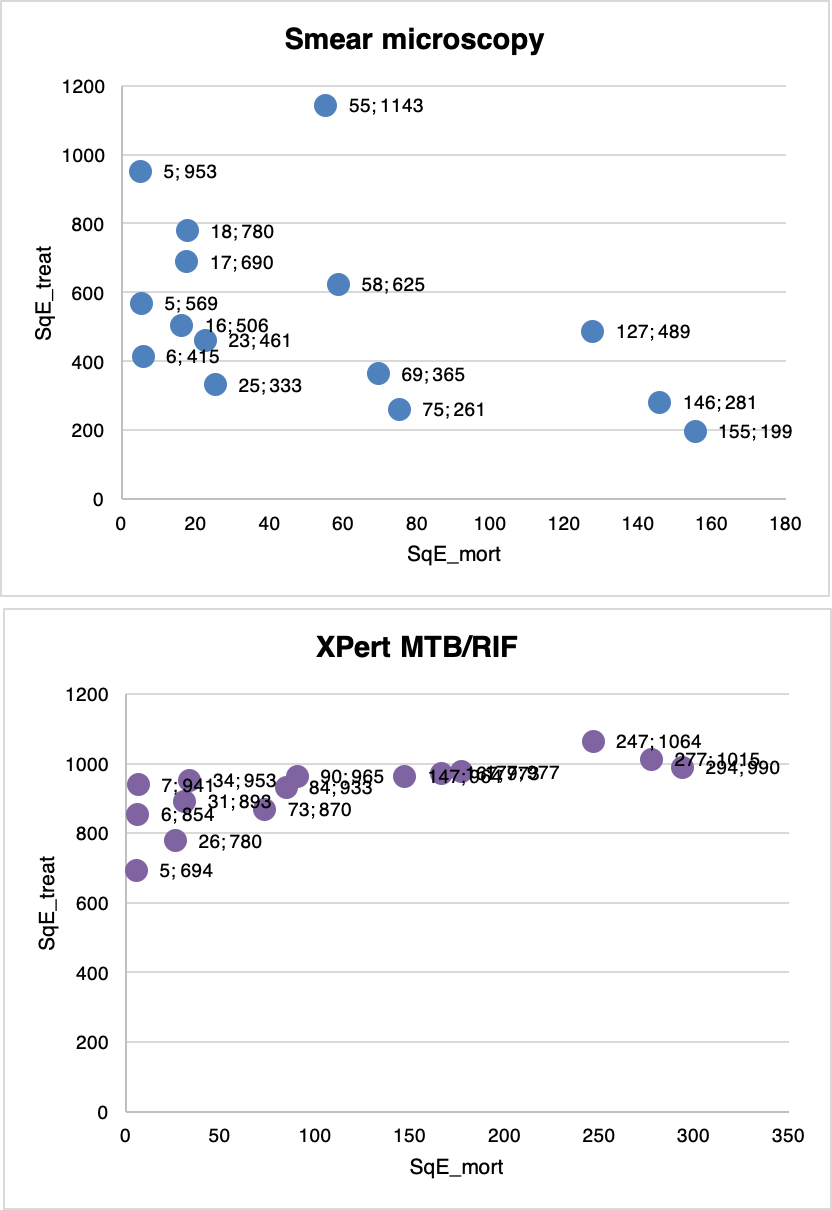


Figure 16. Degree of fit, identifying best fit of the graphs based on both mortality and TB treatment started.

In the figure, SqE_treat represents the square root of the error (SqE) of the difference between observed and modelled representations of the time to treatment curve; and SqE_mort represents the square root of the error (SqE) of the difference between observed and modelled representation of the time to mortality curve.

In the control arm of the analysis, sputum microscopy, the best fit was at the point where health care workers had a 0.25 probability of making the correct decision, with a prevalence of extra-pulmonary TB of approximately 2%. In contrast, the fit of the Xpert arm of the trial estimated a 0% prevalence of extra-pulmonary TB and a probability of 1 of making the correct decision. This result is supported by other analyses of the trial data, that found that the Xpert arm of the trial was slightly healthier than the microscopy/ control arm and that health care workers were more likely to conduct further diagnostic tests and start treatment empirically in the microscopy arm of the trial than in the control arm (8,35,36). The unobserved transition probabilities estimated during the calibration and used in the analyses are summarised in Table 11.

The baseline characteristics of the cohort in the arms of the trial were slightly imbalanced as described above. The model was adjusted for the imbalance in patient population between the two arms of the trial, by implementing the following changes. The calibration modification to account for healthier patients in the smear arm of the trial:

- remove calibration modification (50% mortality reduction for those HIV positive) for healthier patients in the Xpert arm
- same proportion of HIV and ART types of individuals through the model
- recalculate (using the 2x2 tables) the probabilities of having a positive test result and starting treatment based on an equivalent bacteriologically confirmed TB prevalence between the two arms

# **Economic analyses**

Costs incurred by the cohort simulated in the model is estimated by adding a unit cost value multiplied by the utilisation associated with a specific health state or process during the specified time interval. Costs were assessed from a societal perspective and are reported in 2013 US dollars (USD). Using similar arguments to ones posed by Meyer-Rath et al. 2015, costs were not inflated to present values (37), because adjusting for inflation would not accurately represent the present value of resource inputs given that some of the inputs do not track the consumer price inflation (CPI) index. For example, the prices of medicines used in the public sector are decided through a tendering process and then set for a number of years (38). Notably, human resource costs, the main driver of most of these unit costs are negotiated with labour unions, during this negotiation, the trajectory of increases are set.

Unit costs were estimated as part of primary data collection alongside the trial, sampled from the same study sites where the outcomes data were collected. The details of the methodology associated with these costing studies have been published (27,39–41). A combination of top-down and bottom-up costing methods were used. So, for example, facility overhead costs were allocated to specific processes using a utilisation or staff time allocation factor. Processes were observed, inputs noted and valued, and interactions timed to estimate the unit cost of a procedure or input (40).

Provider costs, the cost of diagnosing and treating patients with TB, were estimated for eight of the primary health care facilities included in the study (two per province) and included the cost of building health care facilities, the cost of human resources, the cost of any observed resources used and the cost of medication. The cost of medication was estimated from the South African Department of Health medicines price registry, which lists the tender price of medicines negotiated. We added 8% of the tender price of the medicine, to this cost for the distribution system (ref Margaret von Zeil, personal communication). For MDR TB treatment, we followed the estimates of Sinanovic and colleagues who constructed a cost of RR TB treatment by assuming a mixture of centralised and decentralised models of care were used nationally based on a 54%: 46% urban-rural split (41). Inpatient care for MDR treatment was assumed to be 44 days in the fully decentralised model and 128 days in the fully centralised model. The cost associated with Xpert in the laboratory was likewise calculated from primary data collection in twenty laboratories during test implementation, and includes the cost of laboratory space used to process the test, human resource costs (based on time spent processing observed), as well as the cost of any resources needed to conduct the required assays (40). Provider unit costs used are summarised in Table 5.

Table 5. Provider unit costs in 2013 US$.

| **Description** | **Value or calculation** | **Distribution** | **Reference** |
| --- | --- | --- | --- |
| Antibiotic trial (5 days) per patient, drug costs | $0.28 | Uniform | Vassall et al. 2017; Churchyard et al. 2015 |
| Chest x-ray | $15.17 | Uniform | Foster et al. 2017. |
| Culture | $12.90 | Uniform | Cunnama et al. 2016. |
| Culture (DST) | $25.10 | Uniform | Cunnama et al. 2016. |
| Inpatient bed day | $71.61 | Uniform |  |
| LPA | $20.30 | Uniform | Cunnama et al. 2016. |
| Microscopy test | $8.67 | Uniform | Cunnama et al. 2016. |
| Public clinic visit | $12.54 | Uniform | Vassall et al. 2017. |
| Treatment visit | $7.32 | Uniform | Vassall et al. 2017. |
| Xpert test | $16.90 | Uniform | Cunnama et al. 2016. |
| DS treatment, time-dependent | IF((0.1809(_tunnel)^5 – 6.9701(_tunnel)^4 + 73.436(_tunnel)^3 – 309.63(_tunnel)^2 + 528.71(_tunnel) – 246.25)<0;0;0.1809(_tunnel)^5 – 6.9701(_tunnel)^4 + 73.436(_tunnel)^3 – 309.63(_tunnel)^2 + 528.71(_tunnel) – 246.25) | time-varying | Estimated from Vassall et al. 2017. |

In the Table, DS treatment refers to drug-sensitive treatment; LPA line probe assay; DST drug sensitivity test.

The costs incurred by patients were estimated from patient exit interviews conducted with two cohorts of patients, in ten of the XTEND study clinics (27,39). The unit of analysis was the patient within their household and community. The first cohort of 351 people with suspected TB were interviewed at the time of receiving a TB diagnostic test and followed up six months later. The second cohort, 168 patients on TB treatment were recruited from the same ten facilities and followed up at five months on treatment. In addition, 134 RR TB patients at different stages in their treatment were interviewed with 82 of these receiving inpatient care and 52 receiving treatment in outpatient facilities. We estimated health care utilisation, out of pocket costs incurred due to transport and other expenses incurred. We also estimated patients’ income and income loss associated with ill health; as well as the cost of informal care. Cost results are presented separately for patient costs and ‘community costs’ that includes the cost of informal care.

The costs associated with health seeking behaviour and time loss from the start of TB associated symptoms to getting tested for TB were estimated. The number of health service visits associated with receiving health care during case finding and treatment was based on a combination of patient reported (patient surveys), and provider reported visits for each facility.

Where intervention scenarios modelled increased ART uptake, we included a monthly cost of ARV treatment and associated patient costs from secondary data sources. However, we do not include the cost of ART in all comparators. In the trial population, the implementation of Xpert did not increase the proportion of patients starting ART when compared against the smear microscopy arm of the study (8) and it is likely that adding the cost of ART could make interventions that differentially benefit those who are HIV negative appear more cost-effective (due to the significantly lower costs) than interventions that benefit patients on ART, with potential equity implications in the distribution of resources (42).

The use of health services as patients progressed through care was collected as part of the trial through case note abstractions of identified fields in the patient records. In addition, patients were asked specific questions about their use of health services during their illness and care seeking.

A range of outcomes were used in the economic evaluation. The primary outcome from the trial was mortality six months after a TB diagnostic test (8). In the economic evaluation, we were also interested in the process or intermediary outcomes, these included the number of patients started on treatment; bacteriologically confirmed TB; initial loss-to-follow-up; started on drug-sensitive TB treatment; started on retreatment; and started on MDR-TB treatment. In addition, the proportion of patients with true TB was estimated using the model and reported in the results. The primary outcome of the economic evaluation was disability-adjusted life years averted (DALYs), based on global recommendations for comparability across interventions (43). DALYs were estimated by adding years of life diseased (YLD) to the years of life lost (YLL). YLD is estimated by multiplying the number of cases by the disease duration and the disability weights. Standard disability weights from the Global Burden of Disease study were used. YLL was estimated by multiplying the number of deaths by the life expectancy at the age of death (in other words the number of years lost due to premature mortality). For the average age of onset of TB, the average age of the TB cohort interviewed (38.16 years) was used. Average South African life expectancy at birth of 63 years^[[1]](#footnote-1)^ was used (44). We assumed that disability weights are not additive but used the highest disability weight were more than one disease was present.

# **Sensitivity and scenario analyses**

Univariate sensitivity analyses were conducted, whereby each individual variable in the model is varied downwards by a factor of 10, to provide a low estimate; and upwards by a factor of 10 to provide a high estimate of the outcomes of interest, namely provider costs, societal costs and disability adjusted life years (DALYs).

Table 6. Univariate sensitivity analysis, provider perspective (base: $89.37), societal perspective (base: $168.80), and effectiveness (base: 4.64 DALYs) with the top ten contributors to variability highlighted.

|  |  |  | **Provider perspective (Base: $89.37)** | | | **Societal perspective(Base: $168.80)** | | | **Effectiveness (Base: 4.64 DALYs)** | | |
| --- | --- | --- | --- | --- | --- | --- | --- | --- | --- | --- | --- |
| **Parameter description** | **Low value** | **High value** | **Cost_low value** | **Cost_high value** | **% var** | **Cost_low value** | **Cost_high value** | **% var** | **DALYs_low value** | **DALYs_high value** | **% var** |
| Provider cost of DS treatment, monthly | 3 | 309 | 70 | 282 | 26% | 150 | 362 | 3% | 4.64 | 4.64 | 0% |
| Provider cost of MDR treatment, monthly | 52 | 5168 | 73 | 251 | 18% | 153 | 330 | 2% | 4.64 | 4.64 | 0% |
| Provider cost of Xpert test | 2 | 169 | 74 | 247 | 17% | 153 | 326 | 2% | 4.64 | 4.64 | 0% |
| Provider cost of a clinic visit | 1 | 86 | 75 | 232 | 14% | 155 | 311 | 2% | 4.64 | 4.64 | 0% |
| Prob of a positive TB test result, HIV positive | 0 | 1 | 76 | 178 | 6% | 126 | 447 | 7% | 4.27 | 7.05 | 5% |
| Prob of a positive TB test result, HIV negative | 0 | 1 | 81 | 178 | 5% | 146 | 394 | 4% | 4.62 | 4.89 | 0% |
| Provider cost of chest x-ray | 2 | 152 | 83 | 153 | 3% | 162 | 233 | 0% | 4.64 | 4.64 | 0% |
| Prop of cohort who get a chest x-ray after a negative test result | 0 | 5 | 83 | 152 | 3% | 163 | 232 | 0% | 4.64 | 4.64 | 0% |
| Prob of starting MDR-TB treatment after a positive result, if HIV negative | 0 | 0 | 84 | 143 | 2% | 161 | 246 | 0% | 4.64 | 4.65 | 0% |
| Prob of starting MDR-TB treatment after a positive result, if HIV positive | 0 | 0 | 84 | 142 | 2% | 162 | 242 | 0% | 4.63 | 4.76 | 0% |
| Provider cost of a LPA test | 2 | 203 | 85 | 133 | 1% | 164 | 213 | 0% | 4.64 | 4.64 | 0% |
| Prob of starting MDR-TB treatment from out-of-care, if HIV positive on ART | 0 | 1 | 84 | 117 | 1% | 162 | 209 | 0% | 4.64 | 4.64 | 0% |
| Prop of cohort who access a clinic after a negative test result, if HIV positive | 0 | 7 | 87 | 115 | 0% | 166 | 194 | 0% | 4.64 | 4.64 | 0% |
| Prob of starting MDR-TB treatment after positive result, if HIV positive on ART | 0 | 0 | 89 | 115 | 0% | 169 | 206 | 0% | 4.64 | 4.65 | 0% |
| Prop of cohort who get a DST for Rif resistance after a negative result | 0 | 1 | 87 | 108 | 0% | 167 | 188 | 0% | 4.64 | 4.64 | 0% |
| Provider cost of a hospital bed day | 7 | 716 | 88 | 106 | 0% | 167 | 185 | 0% | 4.64 | 4.64 | 0% |
| Prop of cohort who access a clinic after a negative test result, if HIV negative | 0 | 7 | 88 | 105 | 0% | 167 | 184 | 0% | 4.64 | 4.64 | 0% |
| Prob of a positive TB test result, HIV positive on ART | 0 | 1 | 87 | 104 | 0% | 156 | 247 | 1% | 4.50 | 4.66 | 0% |
| Prob of starting MDR-TB treatment from out-of-care, if HIV positive | 0 | 0 | 89 | 106 | 0% | 169 | 192 | 0% | 4.64 | 4.65 | 0% |
| Prop of cohort, HIV positive on ART | 0 | 0 | 84 | 100 | 0% | 158 | 193 | 0% | 4.31 | 5.36 | 1% |
| Prob of starting TB treatment after a positive test result, HIV positive with TB | 0 | 1 | 76 | 91 | 0% | 128 | 173 | 0% | 4.54 | 5.81 | 1% |
| Prop of cohort who get a DST for INH resistance after a negative test result | 0 | 1 | 88 | 102 | 0% | 168 | 181 | 0% | 4.64 | 4.64 | 0% |
| Provider cost of a smear microscopy test, fluoro-microscopy | 1 | 63 | 88 | 101 | 0% | 168 | 181 | 0% | 4.64 | 4.64 | 0% |
| Provider cost of a culture test | 1 | 129 | 88 | 100 | 0% | 168 | 180 | 0% | 4.64 | 4.64 | 0% |
| Prop of cohort who get a smear microscopy test after a negative test result | 0 | 2 | 88 | 100 | 0% | 168 | 179 | 0% | 4.64 | 4.64 | 0% |
| Prop of cohort who access a clinic after a negative test result, if HIV positive ART | 0 | 10 | 88 | 99 | 0% | 168 | 179 | 0% | 4.64 | 4.64 | 0% |
| Prop of cohort hospitalised after a positive test result, HIV positive | 0 | 2 | 89 | 99 | 0% | 168 | 178 | 0% | 4.64 | 4.64 | 0% |
| Prop of cohort who access a clinic after a positive test result, HIV positive | 0 | 16 | 88 | 98 | 0% | 168 | 178 | 0% | 4.64 | 4.64 | 0% |
| Prop of cohort who get a culture test after negative test result | 0 | 1 | 89 | 97 | 0% | 168 | 177 | 0% | 4.64 | 4.64 | 0% |
| Prob of starting TB treatment after a positive test result, HIV negative with TB | 0 | 1 | 82 | 89 | 0% | 149 | 168 | 0% | 4.65 | 4.89 | 0% |
| Prop of cohort hospitalised after a negative test result, HIV positive on ART | 0 | 1 | 89 | 95 | 0% | 168 | 175 | 0% | 4.64 | 4.64 | 0% |
| Prob of starting the negative pathway after a negative test result, HIV positive | 0 | 0 | 89 | 95 | 0% | 168 | 174 | 0% | 4.64 | 4.64 | 0% |
| Prop of cohort hospitalised after a negative test result, HIV positive | 0 | 0 | 89 | 94 | 0% | 168 | 174 | 0% | 4.64 | 4.64 | 0% |
| Prop of cohort who get a DST for Rif resistance after a positive test result | 0 | 2 | 89 | 93 | 0% | 168 | 173 | 0% | 4.64 | 4.64 | 0% |
| Prop of cohort who get a second Xpert test as part of the negative pathway | 0 | 9 | 89 | 93 | 0% | 168 | 173 | 0% | 4.64 | 4.64 | 0% |
| Prob of TB from a positive test result, HIV positive | 0 | 1 | 85 | 89 | 0% | 165 | 169 | 0% | 3.95 | 4.65 | 0% |
| Prop of cohort who get a DST for INH resistance as part of the negative pathway | 0 | 7 | 89 | 93 | 0% | 168 | 172 | 0% | 4.64 | 4.64 | 0% |
| Prop of cohort who get a DST for Rif resistance as part of the negative pathway | 0 | 7 | 89 | 93 | 0% | 168 | 172 | 0% | 4.64 | 4.64 | 0% |
| Prop of cohort hospitalised after a positive test result, HIV negative | 0 | 2 | 89 | 93 | 0% | 169 | 172 | 0% | 4.64 | 4.64 | 0% |
| Prop of cohort hospitalised after a positive test result, HIV positive on ART | 0 | 2 | 89 | 92 | 0% | 169 | 172 | 0% | 4.64 | 4.64 | 0% |
| Prop of cohort hospitalised as part of the negative pathway, HIV positive | 0 | 2 | 89 | 92 | 0% | 169 | 172 | 0% | 4.64 | 4.64 | 0% |
| Prob of starting TB treatment after a positive test result, HIV positive with TB | 0 | 1 | 87 | 89 | 0% | 157 | 169 | 0% | 4.63 | 5.03 | 0% |
| Prop of cohort who get a culture test as part of the negative pathway | 0 | 7 | 89 | 92 | 0% | 169 | 171 | 0% | 4.64 | 4.64 | 0% |
| Prob of starting treatment from the negative pathway, HIV positive no TB | 0 | 1 | 89 | 92 | 0% | 169 | 179 | 0% | 4.61 | 4.64 | 0% |
| Prop of cohort who access a clinic after a positive test result, HIV negative | 0 | 11 | 89 | 91 | 0% | 169 | 171 | 0% | 4.64 | 4.64 | 0% |
| Probablity of starting treatment after a negative test result, HIV positive | 0 | 0 | 89 | 91 | 0% | 168 | 177 | 0% | 4.62 | 4.64 | 0% |
| Prob of starting the negative pathway after a negative test result, HIV positive ART | 0 | 1 | 89 | 91 | 0% | 169 | 171 | 0% | 4.64 | 4.64 | 0% |
| Prop of cohort, HIV positive | 0 | 1 | 88 | 90 | 0% | 157 | 176 | 0% | 2.02 | 6.31 | 11% |
| Prop of cohort who get a DST for INH resistance after a positive test result | 0 | 1 | 89 | 91 | 0% | 169 | 171 | 0% | 4.64 | 4.64 | 0% |
| Prop of cohort who get a smear microscopy test after a positive test result | 0 | 3 | 89 | 91 | 0% | 169 | 171 | 0% | 4.64 | 4.64 | 0% |
| Prop of cohort who access a clinic after a positive test result, HIV positive on ART | 0 | 11 | 89 | 91 | 0% | 169 | 171 | 0% | 4.64 | 4.64 | 0% |
| Prop of cohort starting MDR-TB treatment after negative test result, HIV positive | 0 | 0 | 89 | 91 | 0% | 169 | 171 | 0% | 4.64 | 4.64 | 0% |
| Prop of cohort who get a chest x-ray as part of the negative pathway | 0 | 4 | 89 | 91 | 0% | 169 | 171 | 0% | 4.64 | 4.64 | 0% |
| Provider cost of an antibiotic trial | 0 | 3 | 89 | 91 | 0% | 169 | 170 | 0% | 4.64 | 4.64 | 0% |
| Prop of cohort hospitalised after a negative test result, HIV negative | 0 | 0 | 89 | 91 | 0% | 169 | 170 | 0% | 4.64 | 4.64 | 0% |
| Prop of cohort who get an antibiotic trial after a negative test result | 0 | 6 | 89 | 91 | 0% | 169 | 170 | 0% | 4.64 | 4.64 | 0% |
| Prop of cohort hospitalised as part of the negative pathway, HIV positive on ART | 0 | 2 | 89 | 91 | 0% | 169 | 170 | 0% | 4.64 | 4.64 | 0% |
| Prop of cohort who get a chest x-ray after a positive test result | 0 | 1 | 89 | 90 | 0% | 169 | 170 | 0% | 4.64 | 4.64 | 0% |
| Prob of starting TB treatment after a positive test result, HIV negative | 0 | 1 | 88 | 89 | 0% | 166 | 169 | 0% | 4.64 | 4.64 | 0% |
| Prop of cohort who get a second Xpert test after a positive test result | 0 | 1 | 89 | 90 | 0% | 169 | 170 | 0% | 4.64 | 4.64 | 0% |
| Prop of cohort who access a clinic as part of the negative pathway, HIV positive | 0 | 6 | 89 | 90 | 0% | 169 | 170 | 0% | 4.64 | 4.64 | 0% |
| Prob of starting the negative pathway after a negative test result, HIV positive with TB | 0 | 0 | 89 | 90 | 0% | 169 | 171 | 0% | 4.60 | 4.65 | 0% |
| Prob of starting TB treatment after a negative test result, HIV positive with TB | 0 | 0 | 89 | 90 | 0% | 168 | 172 | 0% | 4.51 | 4.66 | 0% |
| Prop of cohort who get a culture test after a positive test result | 0 | 1 | 89 | 90 | 0% | 169 | 170 | 0% | 4.64 | 4.64 | 0% |
| Prop of cohort who get a second Xpert test after a negative test result | 0 | 0 | 89 | 90 | 0% | 169 | 170 | 0% | 4.64 | 4.64 | 0% |
| Probabiility of hard-to-diagnose TB in those with a negative TB test result, HIV positive on ART | 0 | 1 | 89 | 89 | 0% | 168 | 169 | 0% | 4.43 | 6.74 | 3% |
| Prob of starting TB treatment after a positive test result, HIV positive | 0 | 1 | 89 | 89 | 0% | 166 | 169 | 0% | 4.64 | 4.65 | 0% |
| Prob of TB from a negative TB test result, HIV positive on ART | 0 | 0 | 89 | 89 | 0% | 168 | 169 | 0% | 4.53 | 5.73 | 1% |
| Prob of mortality from out of care, HIV positive on ART with TB | 0 | 0 | 89 | 89 | 0% | 168 | 169 | 0% | 4.36 | 4.78 | 0% |
| Prob of starting the negative pathway after a negative test result, HIV positive ART with TB | 0 | 1 | 89 | 90 | 0% | 169 | 169 | 0% | 4.63 | 4.64 | 0% |
| Prop of cohort who access a clinic as part of the negative pathway, HIV positive on ART | 0 | 4 | 89 | 90 | 0% | 169 | 169 | 0% | 4.64 | 4.64 | 0% |
| Prop of cohort who get a smear microscopy test as part of the negative pathway | 0 | 2 | 89 | 90 | 0% | 169 | 169 | 0% | 4.64 | 4.64 | 0% |
| Prob of TB from a positive test result, HIV negative | 0 | 1 | 89 | 90 | 0% | 169 | 169 | 0% | 4.60 | 4.65 | 0% |
| Probabiility of hard-to-diagnose TB in those with a negative TB test result, HIV positive | 0 | 1 | 89 | 89 | 0% | 168 | 169 | 0% | 3.74 | 13.77 | 59% |
| Prop of cohort who get a INH pheno test adter a negative test result | 0 | 0 | 89 | 90 | 0% | 169 | 169 | 0% | 4.64 | 4.64 | 0% |
| Prop of cohort who get a RIF pheno test after a negative test result | 0 | 0 | 89 | 90 | 0% | 169 | 169 | 0% | 4.64 | 4.64 | 0% |
| Prob of starting TB treatment after a negative test result, HIV positive on ART no TB | 0 | 0 | 89 | 90 | 0% | 169 | 170 | 0% | 4.64 | 4.64 | 0% |
| Prob of starting TB treatment after a positive test result, HIV positive on ART | 0 | 1 | 89 | 89 | 0% | 168 | 169 | 0% | 4.64 | 4.64 | 0% |
| Prob of TB from a negative TB test result, HIV positive | 0 | 0 | 89 | 89 | 0% | 168 | 169 | 0% | 4.18 | 9.27 | 15% |
| Prop of cohort who get an antibiotic trial after a positive test result | 0 | 4 | 89 | 89 | 0% | 169 | 169 | 0% | 4.64 | 4.64 | 0% |
| Prop of cohort who get a INH pheno test as part of the negative pathway | 0 | 3 | 89 | 89 | 0% | 169 | 169 | 0% | 4.64 | 4.64 | 0% |
| Prop of cohort who get a RIF pheno test as part of the negative pathway | 0 | 3 | 89 | 89 | 0% | 169 | 169 | 0% | 4.64 | 4.64 | 0% |
| Prob of starting TB treatment following the negative pathway, HIV positive on ART with TB | 0 | 1 | 89 | 89 | 0% | 169 | 169 | 0% | 4.63 | 4.64 | 0% |
| Prob of TB from a positive test result, HIV positive on ART | 0 | 1 | 89 | 89 | 0% | 169 | 169 | 0% | 4.60 | 4.64 | 0% |
| Prop of cohort who get a INH pheno test after a positive test result | 0 | 1 | 89 | 89 | 0% | 169 | 169 | 0% | 4.64 | 4.64 | 0% |
| Prob of mortality (all cause) if no TB, HIV positive on ART | 0 | 0 | 89 | 89 | 0% | 169 | 169 | 0% | 4.64 | 4.68 | 0% |
| Prop of cohort who get an antibiotic trial as part of the negative pathway | 0 | 7 | 89 | 89 | 0% | 169 | 169 | 0% | 4.64 | 4.64 | 0% |
| Prob of mortality (all cause) if no TB, HIV positive | 0 | 0 | 89 | 89 | 0% | 169 | 169 | 0% | 4.62 | 4.87 | 0% |
| Prob of mortality with TB, HIV positive | 0 | 0 | 89 | 89 | 0% | 169 | 169 | 0% | 4.63 | 4.65 | 0% |
| Prob of starting TB treatment after a negative test result, HIV positive on ART with TB | 0 | 0 | 89 | 89 | 0% | 169 | 169 | 0% | 4.64 | 4.64 | 0% |
| Prob of mortality with TB, HIV positive on ART | 0 | 0 | 89 | 89 | 0% | 169 | 169 | 0% | 4.64 | 4.65 | 0% |
| Prop of cohort who get a RIF pheno test after a positive test result | 0 | 0 | 89 | 89 | 0% | 169 | 169 | 0% | 4.64 | 4.64 | 0% |
| Prob of TB from a negative TB test result, HIV negative | 0 | 0 | 89 | 89 | 0% | 169 | 169 | 0% | 4.60 | 5.09 | 0% |
| Prob of starting TB treatment following the negative pathway, HIV positive on ART no TB | 0 | 0 | 89 | 89 | 0% | 169 | 169 | 0% | 4.64 | 4.64 | 0% |
| Prob of mortality with TB, HIV negative | 0 | 0 | 89 | 89 | 0% | 169 | 169 | 0% | 4.64 | 4.68 | 0% |
| Prob of starting TB treatment following the negative pathway, HIV positive with TB | 0 | 0 | 89 | 89 | 0% | 169 | 169 | 0% | 4.65 | 4.65 | 0% |
| Prob of mortality (all cause) if no TB, HIV negative | 0 | 0 | 89 | 89 | 0% | 169 | 169 | 0% | 4.64 | 4.65 | 0% |
| Prob of mortality (all cause) if no TB, HIV positive on ART | 0 | 0 | 89 | 89 | 0% | 169 | 169 | 0% | 4.64 | 4.64 | 0% |
| Prob of mortality (all cause) if no TB, HIV positive | 0 | 0 | 89 | 89 | 0% | 169 | 169 | 0% | 4.64 | 4.64 | 0% |
| Provider cost of anti-retroviral therapy, year one | 9 | 940 | 89 | 89 | 0% | 169 | 169 | 0% | 4.64 | 4.64 | 0% |
| Provider cost of anti-retroviral therapy, year two | 5 | 524 | 89 | 89 | 0% | 169 | 169 | 0% | 4.64 | 4.64 | 0% |
| Patient cost of a clinic visit | 1 | 52 | 89 | 89 | 0% | 169 | 169 | 0% | 4.64 | 4.64 | 0% |
| Patient cost of a hospital bed day | 0 | 4 | 89 | 89 | 0% | 169 | 169 | 0% | 4.64 | 4.64 | 0% |
| Patient cost of a hospital bed day, per night | 0 | 4 | 89 | 89 | 0% | 169 | 169 | 0% | 4.64 | 4.64 | 0% |
| Patient cost of a hospital visit, per visit | 6 | 580 | 89 | 89 | 0% | 169 | 169 | 0% | 4.64 | 4.64 | 0% |
| Patient cost fof a visit to a private health facility, per visit | 3 | 292 | 89 | 89 | 0% | 169 | 169 | 0% | 4.64 | 4.64 | 0% |
| Patient cost of a clinic visit, per visit | 0 | 29 | 89 | 89 | 0% | 169 | 169 | 0% | 4.64 | 4.64 | 0% |
| Patient cost of nutritional supplements, per day | 0 | 3 | 89 | 89 | 0% | 169 | 169 | 0% | 4.64 | 4.64 | 0% |
| Disability weights, HIV positive on ART with no TB | 0 | 1 | 89 | 89 | 0% | 169 | 169 | 0% | 4.63 | 4.80 | 0% |
| Disability weights, HIV positive on ART with TB | 0 | 3 | 89 | 89 | 0% | 169 | 169 | 0% | 4.63 | 4.75 | 0% |
| Disability weights, HIV positive with no TB | 0 | 2 | 89 | 89 | 0% | 169 | 169 | 0% | 4.42 | 6.90 | 4% |
| Disability weights, HIV positive with TB | 0 | 4 | 89 | 89 | 0% | 169 | 169 | 0% | 4.62 | 4.88 | 0% |
| Disability weights, HIV negative with TB | 0 | 3 | 89 | 89 | 0% | 169 | 169 | 0% | 4.64 | 4.71 | 0% |
| Prop of cohort on DS-TB treatment (continuation phase), monthly | 0 | 10 | 89 | 89 | 0% | 169 | 169 | 0% | 4.64 | 4.64 | 0% |
| Prop of cohort on DS-TB treatment (intensive phase), monthly | 0 | 40 | 89 | 89 | 0% | 169 | 169 | 0% | 4.64 | 4.64 | 0% |
| Prop of cohort who get a follow-up Xpert test while on treatment | 0 | 1 | 89 | 89 | 0% | 169 | 169 | 0% | 4.64 | 4.64 | 0% |
| Prop of cohort who get a DST for Rif resistance while on treatment | 0 | 1 | 89 | 89 | 0% | 169 | 169 | 0% | 4.64 | 4.64 | 0% |
| Prop of cohort who get a RIF pheno test | 0 | 0 | 89 | 89 | 0% | 169 | 169 | 0% | 4.64 | 4.64 | 0% |
| Prop of cohort who get a smear microscopy test during TB treatment | 0 | 11 | 89 | 89 | 0% | 169 | 169 | 0% | 4.64 | 4.64 | 0% |
| Prop of cohort who access a private faclity after a negative test result, HIV negative | 0 | 10 | 89 | 89 | 0% | 169 | 169 | 0% | 4.64 | 4.64 | 0% |
| Prop of cohort who access a private facility after a negative test result, HIV positive | 0 | 2 | 89 | 89 | 0% | 169 | 169 | 0% | 4.64 | 4.64 | 0% |
| Prop of cohort who access a private facility after a negative test result, HIV positive on ART | 0 | 1 | 89 | 89 | 0% | 169 | 169 | 0% | 4.64 | 4.64 | 0% |
| Prop of cohort who access a private facility as part of the negative pathway, HIV positive | 0 | 1 | 89 | 89 | 0% | 169 | 169 | 0% | 4.64 | 4.64 | 0% |
| Prop of cohort who access a private facility as part of the negative pathway, HIV positive ART | 0 | 1 | 89 | 89 | 0% | 169 | 169 | 0% | 4.64 | 4.64 | 0% |
| Prop of cohort who access a private facility after a positive test result, HIV negative | 0 | 2 | 89 | 89 | 0% | 169 | 169 | 0% | 4.64 | 4.64 | 0% |
| Prop of cohort who access a private facility after a positive test result, HIV positive | 0 | 1 | 89 | 89 | 0% | 169 | 169 | 0% | 4.64 | 4.64 | 0% |
| Prop of cohort who access a private facility after a positive test result, HIV positive on ART | 0 | 3 | 89 | 89 | 0% | 169 | 169 | 0% | 4.64 | 4.64 | 0% |
| Prop of cohort starting MDR-TB treatment after negative test result, HIV negative | 0 | 0 | 89 | 89 | 0% | 169 | 169 | 0% | 4.64 | 4.64 | 0% |
| Prop of cohort starting MDR-TB treatment after a negative test result, HIV positive on ART | 0 | 0 | 89 | 89 | 0% | 169 | 169 | 0% | 4.64 | 4.64 | 0% |
| Prop of cohort starting MDR-TB after being out-of-care, HIV negative | 0 | 0 | 89 | 89 | 0% | 169 | 169 | 0% | 4.64 | 4.64 | 0% |
| Provider cost of DS-TB treatment (time dependent function), monthly | 4 | 395 | 89 | 89 | 0% | 169 | 169 | 0% | 4.64 | 4.64 | 0% |
| Patient cost of accessing DS-TB treatment, time dependent function | 16 | 1557 | 89 | 89 | 0% | 125 | 1195 | 73% | 4.64 | 4.64 | 0% |
| Patient cost of accessing MDR-TB treatment, time dependent function | 19 | 1881 | 89 | 89 | 0% | 164 | 229 | 0% | 4.64 | 4.64 | 0% |
| Patient cost of illness, while on DS-TB treatment, time dependent function | 4 | 409 | 89 | 89 | 0% | 156 | 436 | 5% | 4.64 | 4.64 | 0% |
| Patient cost of illness, while on MDR-TB treatment, time dependent function | 3 | 329 | 89 | 89 | 0% | 166 | 177 | 0% | 4.64 | 4.64 | 0% |
| Prob of starting TB treatment after a negative test result, HIV negative no TB | 0 | 0 | 89 | 89 | 0% | 169 | 169 | 0% | 4.64 | 4.64 | 0% |
| Prob of starting TB treatment after a negative test result, HIV negative with TB | 0 | 0 | 89 | 89 | 0% | 169 | 169 | 0% | 4.64 | 4.64 | 0% |
| Discount rate | 0 | 0 | 88 | 90 | 0% | 165 | 171 | 0% | 4.40 | 4.76 | 0% |

In the Table, prop refers to the proportion of the population, while prob is the probability of an event occurring.

Univariate sensitivity analyses, while useful to identify key drivers of our predicted estimates, have limited explanatory utility in identifying model assumptions that will alter the decision to choose one investment scenario over another. This is partly because multiple variables interact along a decision pathway to produce a cost or an effect, so for example, increasing the value of the unit cost of a chest x-ray is unlikely to be identified as a key driver of the results if a low proportion of the cohort is identified as needing additional diagnostic tests after an initial negative TB test result. These issues are correlated as healthcare workers’ decisions to follow-up a patient after a negative TB test result will be influenced by the availability of chest x-ray facilities, a possible investment strategy. Complementary to univariate sensitivity analyses, scenario analyses are therefore used further explore drivers of the investment decision, by varying multiple parameters simultaneously.

Decision points in the TB diagnostic pathway were identified, along with the downstream decision points that they in turn will influence. The costs incurred, or the costs averted by the interaction at each of the decision points are called the transaction cost (45). The decision points are places in the pathway where health care workers and patients have an interaction, or where alternative courses of action that influences TB outcomes (particularly mortality) occur. These were identified based on a combination of the clinical guidelines used in health facilities, the movement through care captured in the pragmatic cluster randomised control study, and an understanding of the range factors that influences these decisions based on the observations done by the researchers while collecting data for the primary cost analyses (27,39,46). We explore how alternative patterns of behaviour will affect the value of the TB diagnostic algorithm in South Africa by varying multiple interacting probabilities in the pathway.

# **CHEERS checklist for economic evaluations of health interventions**

| **Section/item** | **Item No** | **Recommendation** | **Reported on page No/ line No** |
| --- | --- | --- | --- |
| **Title and abstract** | | | |
| Title | 1 | Identify the study as an economic evaluation or use more specific terms such as “cost-effectiveness analysis”, and describe the interventions compared. | Page 1 line 1 - 3 |
| Abstract | 2 | Provide a structured summary of objectives, perspective, setting, methods (including study design and inputs), results (including base case and uncertainty analyses), and conclusions. | Page 2 line 33 - 65 |
| **Introduction** | | | |
| Background and objectives | 3 | Provide an explicit statement of the broader context for the study. | Page 3 line 69 - 82 |
|  |  | Present the study question and its relevance for health policy or practice decisions. | Page 5 line 103 - 109 |
| **Methods** | | | |
| Target population and subgroups | 4 | Describe characteristics of the base case population and subgroups analysed, including why they were chosen. | Page 6 line 114 - 126 |
| Setting and location | 5 | State relevant aspects of the system(s) in which the decision(s) need(s) to be made. | Detailed in S1 text  Page 33 lines 116 - 126 |
| Study perspective | 6 | Describe the perspective of the study and relate this to the costs being evaluated. | Page 6 line 131  Page 6 line 131 - 133 |
| Comparators | 7 | Describe the interventions or strategies being compared and state why they were chosen. | Page 16 line 245 |
| Time horizon | 8 | State the time horizon(s) over which costs and consequences are being evaluated and say why appropriate. | Page 10 line 165 |
| Discount rate | 9 | Report the choice of discount rate(s) used for costs and outcomes and say why appropriate. | Page 19 line 280 |
| Choice of health outcomes | 10 | Describe what outcomes were used as the measure(s) of benefit in the evaluation and their relevance for the type of analysis performed. | Page 18 line 268 |
| Measurement of effectiveness | 11a | *Single study-based estimates:*Describe fully the design features of the single effectiveness study and why the single study was a sufficient source of clinical effectiveness data. | Page 10 line 173 |
|  | 11b | *Synthesis-based estimates*: Describe fully the methods used for identification of included studies and synthesis of clinical effectiveness data. | Page 10 line 174 – 177 |
| Measurement and valuation of preference-based outcomes | 12 | If applicable, describe the population and methods used to elicit preferences for outcomes. | N/A |
| Estimating resources and costs | 13a | *Single study-based economic evaluation:* Describe approaches used to estimate resource use associated with the alternative interventions. Describe primary or secondary research methods for valuing each resource item in terms of its unit cost. Describe any adjustments made to approximate to opportunity costs. | N/A |
|  | 13b | *Model-based economic evaluation:*Describe approaches and data sources used to estimate resource use associated with model health states. Describe primary or secondary research methods for valuing each resource item in terms of its unit cost. Describe any adjustments made to approximate to opportunity costs. | Page 10 lines 173 – 179  Page 13 lines 199 – 214  Page 14 lines 218 – 231  Page 14 lines 235 - 237 |
| Currency, price date, and conversion | 14 | Report the dates of the estimated resource quantities and unit costs. Describe methods for adjusting estimated unit costs to the year of reported costs if necessary. Describe methods for converting costs into a common currency base and the exchange rate. | Page 14 lines 229 - 231 |
| Choice of model | 15 | Describe and give reasons for the specific type of decision-analytical model used. Providing a figure to show model structure is strongly recommended. | Page 10 line 164 |
| Assumptions | 16 | Describe all structural or other assumptions underpinning the decision-analytical model. | Detailed in S1 text  Page 10 lines 178 - 184 |
| Analytical methods | 17 | Describe all analytical methods supporting the evaluation. This could include methods for dealing with skewed, missing, or censored data; extrapolation methods; methods for pooling data; approaches to validate or make adjustments (such as half cycle corrections) to a model; and methods for handling population heterogeneity and uncertainty. | Detailed in S1 text |
| **Results** | | | |
| Study parameters | 18 | Report the values, ranges, references, and, if used, probability distributions for all parameters. Report reasons or sources for distributions used to represent uncertainty where appropriate. Providing a table to show the input values is strongly recommended. | These are summarised in Table 1, page 11  Page 13 line 200  Page 19 lines 277 - 281 |
| Incremental costs and outcomes | 19 | For each intervention, report mean values for the main categories of estimated costs and outcomes of interest, as well as mean differences between the comparator groups. If applicable, report incremental cost-effectiveness ratios. | Page 22 line 332 |
| Characterising uncertainty | 20a | *Single study-based economic evaluation:* Describe the effects of sampling uncertainty for the estimated incremental cost and incremental effectiveness parameters, together with the impact of methodological assumptions (such as discount rate, study perspective). | N/A |
|  | 20b | *Model-based economic evaluation:*Describe the effects on the results of uncertainty for all input parameters, and uncertainty related to the structure of the model and assumptions. | Detailed in S1 text  Page 24 line 370 - 377 |
| Characterising heterogeneity | 21 | If applicable, report differences in costs, outcomes, or cost-effectiveness that can be explained by variations between subgroups of patients with different baseline characteristics or other observed variability in effects that are not reducible by more information. | Contextual page 28, lines 402 - 412 |
| **Discussion** | | | |
| Study findings, limitations, generalisability, and current knowledge | 22 | Summarise key study findings and describe how they support the conclusions reached. Discuss limitations and the generalisability of the findings and how the findings fit with current knowledge. | Page 30 lines 430 – 443  Page 32 lines 490 - 507 |
| **Other** | | | |
| Source of funding | 23 | Describe how the study was funded and the role of the funder in the identification, design, conduct, and reporting of the analysis. Describe other non-monetary sources of support. | Page 34 lines 540 - 547 |
| Conflicts of interest | 24 | Describe any potential for conflict of interest of study contributors in accordance with journal policy. In the absence of a journal policy, we recommend authors comply with International Committee of Medical Journal Editors recommendations. | Page 34 lines 536 - 537 |

# **References**

1. Ataguba JEJ, Akazili J, McIntyre D, Ataguba JEJ, Akazili J, McIntyre D. Socioeconomic-related health inequality in South Africa: evidence from General Household Surveys. Int J Equity Health [Internet]. 2011;10(1):48. Available from: http://www.equityhealthj.com/content/10/1/48

2. Karyadi E, Schultink W, Nelwan RHH, Gross R, Amin Z, Dolmans WM V, et al. Community and International Nutrition Poor Micronutrient Status of Active Pulmonary Tuberculosis Patients in Indonesia 1. 2000;(August):2953–8.

3. Shah S, Whalen C, Kotler DP, Mayanja H, Namale A, Melikian G, et al. Human Nutrition and Metabolism Severity of Human Immunodeficiency Virus Infection Is Associated with Decreased Phase Angle , Fat Mass and Body Cell Mass in Adults with Pulmonary Tuberculosis Infection in Uganda 1. 2001;(August):2843–7.

4. Oni T, Berkowitz N, Kubjane M, Goliath R, Levitt NS, Wilkinson RJ. Trilateral overlap of tuberculosis, diabetes and HIV-1 in a high-burden African setting: implications for TB control. Tuberculosis [Internet]. 2017;50(96841). Available from: http://dx.doi.org/10.1183/13993003.00004-2017

5. Berkowitz N, Okorie A, Goliath R, Levitt N, Wilkinson RJ, Oni T. The prevalence and determinants of active tuberculosis among diabetes patients in Cape Town , South Africa , a high HIV / TB burden setting. Diabetes Res Clin Pract [Internet]. 2018;138:16–25. Available from: https://doi.org/10.1016/j.diabres.2018.01.018

6. Gupta-Wright A, Corbett EL, van Oosterhout JJ, Wilson D, Grint D, Alufandika-Moyo M, et al. Rapid urine-based screening for tuberculosis in HIV-positive patients admitted to hospital in Africa (STAMP): a pragmatic, multicentre, parallel-group, double-blind, randomised controlled trial. Lancet [Internet]. 2018;392(10144):292–301. Available from: https://linkinghub.elsevier.com/retrieve/pii/S0140673618312674

7. Middelkoop K, Mathema B, Myer L, Shashkina E, Whitelaw A, Kaplan G, et al. Transmission of tuberculosis in a south African community with a high prevalence of HIV infection. J Infect Dis. 2015;211(1):53–61.

8. Churchyard GJ, Stevens WS, Mametja LD, McCarthy KM, Chihota V, Nicol MP, et al. Xpert MTB / RIF versus sputum microscopy as the initial diagnostic test for tuberculosis : a cluster-randomised trial embedded in South African roll-out of Xpert MTB / RIF. Lancet Glob Heal [Internet]. 2015;3(8):e450–7. Available from: http://dx.doi.org/10.1016/S2214-109X(15)00100-X

9. Theron G, Zijenah L, Chanda D, Clowes P, Rachow A, Lesosky M, et al. Feasibility, accuracy, and clinical effect of point-of-care XPert MTB/RIF testing for tuberculosis in primary-care settings in Africa: a multicentre, randomised, controlled trial. Lancet [Internet]. 2013 Oct 25 [cited 2013 Nov 6];6736(13):1–14. Available from: http://www.ncbi.nlm.nih.gov/pubmed/24176145

10. Durovni B, Saraceni V, Hof S Van Den, Trajman A. Impact of Replacing Smear Microscopy with Xpert MTB / RIF for Diagnosing Tuberculosis in Brazil : A. 2014;11(12).

11. Lin H-H, Langley I, Mwenda R, Doulla B, Egwaga S, Millington K a, et al. A modelling framework to support the selection and implementation of new tuberculosis diagnostic tools. Int J Tuberc lung Dis [Internet]. 2011 Aug [cited 2012 Mar 9];15(8):996–1004. Available from: http://www.ncbi.nlm.nih.gov/pubmed/21740663

12. Langley I, Lin H-H, Egwaga S, Doulla B, Ku C-C, Murray M, et al. Assessment of the patient, health system, and population effects of Xpert MTB/RIF and alternative diagnostics for tuberculosis in Tanzania: an integrated modelling approach. Lancet Glob Health. 2014 Oct [cited 2014 Sep 26];2(10):e581–91. Available from: http://linkinghub.elsevier.com/retrieve/pii/S2214109X14702918

13. Lin H, Dowdy D, Dye C. The impact of new tuberculosis diagnostics on transmission : why context matters. :1–22.

14. Dowdy DW. Economic analyses of diagnostics for tuberculosis: what’s the point? Expert Rev pharmacoeconomic outcomes. 2012;12(2):137–9.

15. Kendall EA, Schumacher SG, Denkinger CM, Dowdy DW. Estimated clinical impact of the Xpert MTB / RIF Ultra cartridge for diagnosis of pulmonary tuberculosis : A modeling study. 2017;1–20.

16. Dowdy DW, Cattamanchi A, Steingart KR, Pai M. Is Scale-Up Worth It? Challenges in Economic Analysis of Diagnostic Tests for Tuberculosis. PLoS Med [Internet]. 2011 Jul 26 [cited 2011 Jul 28];8(7):e1001063. Available from: http://dx.plos.org/10.1371/journal.pmed.1001063

17. Vassall A, van Kampen S, Sohn H, Michael JS, John KR, den Boon S, et al. Rapid diagnosis of tuberculosis with the Xpert MTB/RIF assay in high burden countries: a cost-effectiveness analysis. Wilson D, editor. PLoS Med [Internet]. 2011 Nov 8 [cited 2011 Nov 9];8(11):e1001120. Available from: http://www.pubmedcentral.nih.gov/articlerender.fcgi?artid=3210757&tool=pmcentrez&rendertype=abstract

18. Menzies N a., Cohen T, Lin H-HH, Murray M, Salomon JA. Population Health Impact and Cost-Effectiveness of Tuberculosis Diagnosis with Xpert MTB/RIF: A Dynamic Simulation and Economic Evaluation. Rosen S, editor. PLoS Med [Internet]. 2012 Nov 20 [cited 2012 Nov 22];9(11):e1001347. Available from: http://dx.plos.org/10.1371/journal.pmed.1001347

19. Dunbar R, Naidoo P, Beyers N, Langley I. Improving rifampicin-resistant tuberculosis diagnosis using Xpert ^®^ MTB/RIF: modelling interventions and costs. Int J Tuberc Lung Dis [Internet]. 2018;22(8):890–8. Available from: http://www.ingentaconnect.com/content/10.5588/ijtld.17.0594

20. Vassall A, Siapka M, Foster N, Cunnama L, Ramma L, Fielding K, et al. Cost-effectiveness of Xpert MTB/RIF for tuberculosis diagnosis in South Africa: a real-world cost analysis and economic evaluation. Lancet Glob Heal. 2017;5(7).

21. Dodd PJ, Pennington JJ, Bronner Murrison L, Dowdy DW. Simple Inclusion of Complex Diagnostic Algorithms in Infectious Disease Models for Economic Evaluation. Med Decis Mak [Internet]. 2018;38(8):930–41. Available from: http://journals.sagepub.com/doi/10.1177/0272989X18807438

22. Briggs A, Sculpher M. An introduction to Markov modelling for economic evaluation. Pharmacoeconomics [Internet]. 1998 Apr;13(4):397–409. Available from: http://www.ncbi.nlm.nih.gov/pubmed/10178664

23. Glasziou PP, Siegel JE, Weeks JC, Pliskin JS, Elstein AS, Weinstein MC. Decision making in health and medicine Integrating evidence and values.

24. Menzies N. Estimating the cost-effectiveness of XPert MTB/RIF: applying a Bayesian calibration approach to a dynamic TB-HIV epidemic model. In: The 34th annual meeting of the society for medical decision making. Johannesburg,South Africa; 2012.

25. Oxlade O, Piatek A, Vincent C, Menzies D. Modeling the impact of tuberculosis interventions on epidemiologic outcomes and health system costs. BMC Public Health [Internet]. 2015;15(1). Available from: http://www.biomedcentral.com/1471-2458/15/141

26. Dunbar R, Naidoo P, Beyers N, Langley I. High laboratory cost predicted per tuberculosis case diagnosed with increased case finding without a triage strategy. Int J Tuberc Lung Dis [Internet]. 2017;21(9):1026–34. Available from: http://www.ingentaconnect.com/content/10.5588/ijtld.17.0156

27. Foster N, Vassall A, Cleary S, Cunnama L, Churchyard G, Sinanovic E. The economic burden of TB diagnosis and treatment in South Africa. Soc Sci Med [Internet]. 2015;130:42–50. Available from: http://linkinghub.elsevier.com/retrieve/pii/S0277953615000726

28. Naidoo P, Dunbar R, Lombard C, du Toit E, Caldwell J, Detjen A, et al. Comparing Tuberculosis Diagnostic Yield in Smear/Culture and Xpert® MTB/RIF-Based Algorithms Using a Non-Randomised Stepped-Wedge Design. PLoS One [Internet]. 2016;11(3):e0150487. Available from: http://www.ncbi.nlm.nih.gov/pubmed/26930400

29. Muennig P, Bounthavong M. Cost-effectiveness analysis in health: a practical approach. Third. San Francisco, United States of America: Wiley; 2016.

30. Vanni T, Karnon J, Madan J, White RG, Edmunds WJ, Foss AM, et al. Calibrating Models in Economic Evaluation. Pharmacoeconomics. 2011;29(1):51–62.

31. Vanni T, Karnon J, Madan J, White RG, Edmunds WJ, Foss AM, et al. Calibrating models in economic evaluation: a seven-step approach. Pharmacoeconomics. 2011;29(1):51–62.

32. Karnon J, Vanni T. Calibrating Models in Economic Evaluation: a comparison of alternative measures of goodness of fit, parameter search strategies and convergence crietria. Pharmacoeconomics. 2011;29(1):51–62.

33. Railsback SF, Grimm V. Agent-based and individual-based modeling: a practical introduction. Princeton University Press; 2012. 1–329 p.

34. Brown S. Medical false positive and false negatives: conditional probability [Internet]. BrownMath.com. 2013 [cited 2016 Mar 13]. Available from: http://brownmath.com/stat/falsepos.htm

35. McCarthy K, Fielding K, Churchyard GJ, Grant AD. Empiric tuberculosis treatment in South African primary health care facilities - For whom, where, when and why: Implications for the development of tuberculosis diagnostic tests. PLoS One. 2018;13(1):1–14.

36. McCarthy KM, Grant AD, Chihota V, Ginindza S, Mvusi L, Churchyard GJ, et al. Implementation and Operational Research: What Happens After a Negative Test for Tuberculosis? Evaluating Adherence to TB Diagnostic Algorithms in South African Primary Health Clinics. J Acquir Immune Defic Syndr. 2016;

37. Meyer-Rath G, Over M, Klein D, Bershteyn A. The Cost and Cost-Effectiveness of Alternative Strategies to Expand Treatment to HIV-Positive South Africans: Scale Economies and Outreach Costs. Ssrn. 2015;(April 2015).

38. Wouters OJ, Sandberg DM, Kanavos PG, Pillay A. The impact of pharmaceutical tendering on prices and market concentration in South Africa over a 14-year period. Soc Sci Med [Internet]. 2019;In press(June 2018):362–70. Available from: https://doi.org/10.1016/j.socscimed.2018.11.029

39. Ramma L, Cox H, Wilkinson L, Foster N, Cunnama L, Vassall A, et al. Patients’ costs associated with seeking and accessing treatment for drug resistant Tuberculosis in South Africa. Int J Tuberc Lung Dis. 2015;19(12).

40. Cunnama L, Sinanovic E, Ramma L, Foster N, Berrie L, Stevens W, et al. The costs of novel TB diagnostics during scale-up: evidence form the roll-out of Xpert MTB/RIF in South Africa. J Health Econ. 2016;January.

41. Sinanovic E, Ramma L, Vassall A, Azevedo V, Wilkinson L, Ndjeka N, et al. Impact of reduced hospitalisation on the cost of treatment for drug-resistant tuberculosis in South Africa. 2015;19(October 2014):172–8.

42. Vassall A. Xpert MTB/RIF for those with HIV; the importance of considering equity. PLOS blogs. 2012;(November):2012–3.

43. NICE International. Methods for Economic Evaluation Project (MEEP) [Internet]. Seattle: Bill and Melinda Gates Foundation; 2014. Available from: https://www.nice.org.uk/about/what-we-do/nice-international/nice-international-projects/methods-for-economic-evaluation-project-and-the-gates-reference-case

44. Dorrington R, Bradshaw D, Laubscher R, Nannan N. Rapid mortality surveillance report 2016. 2018.

45. Williamson OE. Transaction-Cost Economics: The Governance of Contractual Relations. J Law Econ [Internet]. 1979;22(2):233–61. Available from: http://www.journals.uchicago.edu/doi/10.1086/466942

46. Vassall A, Siapka M, Foster N, Cunnama L, Ramma L, Fielding K, et al. Revisitig the cost-effectiveness of Xpert MTB/RIF: lessons learned from South Africa. Lancet Glob Health. 2017;

1. This was estimated by taking the average of the estimated life expectancies for 2012, 2013, 2014, 2015 and 2016 as reported in the SAMRC rapid mortality surveillance report (44). [↑](#footnote-ref-1)
