# Supplementary material for: Strengthening health systems to improve the value of tuberculosis diagnostics in South Africa: A cost and cost-effectiveness analysis
Source: PLoS One. 2021 May 14;16(5):e0251547. doi: 10.1371/journal.pone.0251547 (PMC8121360; doi:10.1371/journal.pone.0251547)
Supplement: Attachment — Submitted filename: response_to_reviewers.docx [file pone.0251547.s003.docx]

**PONE-D-20-18039
Strengthening health systems to improve the value of Tuberculosis diagnostics in South Africa: a cost and cost-effectiveness analysis.**
Nicola Foster, Lucy Cunnama, Kerrigan McCarthy, Lebogang Ramma, Mariana Siapka, Edina Sinanovic, Gavin Churchyard, Katherine Fielding, Alison D Grant, Susan Cleary

***Response to reviewers***

| Thank you for submitting your manuscript to PLOS ONE. After careful consideration, we feel that it has merit but does not fully meet PLOS ONE’s publication criteria as it currently stands. Therefore, we invite you to submit a revised version of the manuscript that addresses the points raised during the review process.  Please submit your revised manuscript. If you will need more time than this to complete your revisions, please reply to this message or contact the journal office at [plosone@plos.org](mailto:plosone@plos.org). When you're ready to submit your revision, log on to <https://www.editorialmanager.com/pone/> and select the 'Submissions Needing Revision' folder to locate your manuscript file.  Please include the following items when submitting your revised manuscript:   - A rebuttal letter that responds to each point raised by the academic editor and reviewer(s). You should upload this letter as a separate file labeled 'Response to Reviewers'. - A marked-up copy of your manuscript that highlights changes made to the original version. You should upload this as a separate file labeled 'Revised Manuscript with Track Changes'. - An unmarked version of your revised paper without tracked changes. You should upload this as a separate file labeled 'Manuscript'.   If you would like to make changes to your financial disclosure, please include your updated statement in your cover letter. Guidelines for resubmitting your figure files are available below the reviewer comments at the end of this letter. | Thank you for reviews of the manuscript. Our revised submission includes the following documents:   - response_to_reviewers.docx - revised_manuscript.docx (*with track changes*) - manuscript.docx (*without track changes*) - figures - S1_Text.docx - S2_Data.xlsx |
| --- | --- |
| Editor:  When submitting your revision, we need you to address these additional requirements.    1. Please ensure that your manuscript meets PLOS ONE's style requirements, including those for file naming. The PLOS ONE style templates can be found at  <https://journals.plos.org/plosone/s/file?id=wjVg/PLOSOne_formatting_sample_main_body.pdf> and  <https://journals.plos.org/plosone/s/file?id=ba62/PLOSOne_formatting_sample_title_authors_affiliations.pdf> | Files have been renamed in accordance with the style requirements.  The manuscript style for main body and title page has been amended in the manuscript. |
| 2. Your ethics statement must appear in the Methods section of your manuscript. If your ethics statement is written in any section besides the Methods, please move it to the Methods section and delete it from any other section. Please also ensure that your ethics statement is included in your manuscript, as the ethics section of your online submission will not be published alongside your manuscript. | Ethics statement has now been placed at the end of the Methods section (pg 16, ln 4). |
| 3. Please ensure that you refer to Figure 2 in your text as, if accepted, production will need this reference to link the reader to the figure. | The within text reference links to Figure 2 have been fixed. (pg 19, ln 15). |
| 4. Please include captions for your Supporting Information files at the end of your manuscript, and update any in-text citations to match accordingly. Please see our Supporting Information guidelines for more information: <http://journals.plos.org/plosone/s/supporting-information>. |  |
| Reviewer #1:  The authors seek to evaluate where the bottleneck is when efforts are put to improve health outcome for TB community. Walking through the journey from TB diagnostics to timely and effective treatment, the authors identified that improving decision making for TB patients who received negative results from the first-round diagnostic tests. This study has its merits in terms of significance for TB community. Please address the following comments of this reviewer: |  |
| 1. The introduction contains ambiguity. For instance, in Page 11 Line 20-21, you cited a reference (Reference 10) and stated that Xpert implementation is cost- and effect-neutral. However, in that reference, the conclusion was proper ineffective implementation of Xpert was the limiting factor to improve outcome. The statement in the current manuscript could cause confusion whether Xpert technology itself is not effective or there lacks infrastructure for full utilization of this technology. | Removed ambiguity on page 5 by correcting the references to make it clearer that the statement “The study concluded that implementation constraints may have mediated the impact of Xpert under programmatic conditions. (7;10)” is a conclusion from the same study. |
| 2. If the false-negative rate of Xpert is not significant, or the portion of patients with both TB and HIV is not significant, how do you justify your conclusion. In addition, it would be helpful to your conclusion if diagnostic capability of Xpert were briefly introduced to exclude this as a variable in your model. | The relationship between the diagnostic capability of Xpert and the study population is discussed in more detail in the supplementary appendix (S1 Text pages 12 and 13), especially how this is included in the model and in the sensitivity analysis. |
| 3. Please provide a clear definition for cost-effectiveness. | Definition added on page 7, line 4: “Cost-effectiveness analysis is a method for examining the change in costs and the change in health outcomes of a given intervention.” |
| 4. Please fix all language issues throughout the manuscript. | The manuscript has been proof-read including by co-authors for whom English is their mother-tongue. |
| Reviewer #2:  Manuscript Review: PONE-D-20-18039  Key Results: In the manuscript the authors attempt to determine the cost effectiveness of implementing further investments that can complement the use of Xpert-MTB-RIF testing in South Africa by building a mathematical model and incorporating variables. The model was used to identify which investment variable would increase cost effectiveness and improve Tuberculosis diagnostics to alleviate disease burden. In addition to monetary values, the authors also included deaths averted and disability-adjusted-life-years (DALYs) in their analysis. After their analysis, the authors found that investing in several different aspects of tuberculosis care is more cost effective than a single investment. Data showed that in symptomatic patients, costs were only marginally reduced by improving loss to follow-up and a greater good can be achieved by improving access to further testing. Overall, the authors suggest that an investment in the strengthening of the healthcare system’s tuberculosis diagnostics may be an important place to concentrate funds and not just investments in detection.  Validity: Based on the methodology used in this study, I find the manuscript to be valid.  Originality and Significance: I find the data presented in this study to be original and significant. Although what the authors suggest will be difficult to do and will face a lot of red tape, in theory, it is good data to have available.  Data and Methodology: This manuscript presented a straightforward data analysis based on data collected through analysis of a tuberculosis cohort in South Africa with a 13% positive rate. The methodology presented is commonly used and appropriate.  Appropriate us of Statistics: Appropriate statistics were used throughout the study. Proper statistics were conducted, and variables were adjusted for when needed.   Conclusions: Based on the statistics presented in the study, the conclusions appear to be valid and reliable.  Suggested Improvements: I did not find much improvement needed with the manuscript as a whole. The only issue was the sections where there was an error in the references. For example, page 20 lines 15 and 19. These need to be addressed.  References: The references are valid.  Clarity and context: The abstract, introduction and conclusions are clear, concise and appropriate.  Scope of expertise: This manuscript is within the scope of my expertise. | Corrected the error in links to Figure 2 (pg 19, ln 15). |
